# Effect of statins and ACE inhibitors on the metabolome in the Adolescent Type 1 Diabetes cardio-renal Intervention Trial (AdDIT) cohort

**DOI:** 10.1038/s41598-026-50044-w

**Published:** 2026-04-29

**Authors:** Beatrice Prampolini, Antoine Giton, Sofia Frassineti, Paul Benitez-Aguirre, Fergus J. Cameron, Maria E. Craig, Jennifer J. Couper, R. Neil Dalton, Denis Daneman, Elizabeth A. Davis, Kim C. Donaghue, Tim W. Jones, Farid H. Mahmud, Sally M. Marshall, H. Andrew Neil, John E. Deanfield, M. Loredana Marcovecchio, Scott T. Chiesa

**Affiliations:** 1 Department of Medical and Surgical Sciences for Mothers, Children and Adults - Post-Graduate School of Pediatrics, University of Modena and Reggio Emilia, Modena, Italy; 2 Institute of Cardiovascular Science, University College London, London, UK; 3 Institute of Endocrinology and Diabetes, The Children’s Hospital at Westmead, Sydney, Australia; 4 Discipline of Child and Adolescent Health, University of Sydney, Sydney, NSW Australia; 5 Department of Endocrinology and Diabetes, Royal Children’s Hospital, Melbourne, VIC Australia; 6 Murdoch Children’s Research Institute, Melbourne, VIC Australia; 7 The University of Melbourne, Melbourne, VIC Australia; 8 Duke-NUS Medical School, National University of Singapore, Singapore, Singapore; 9 Endocrinology and Diabetes Centre, Women’s and Children’s Hospital, Robinson Institute, University of Adelaide, Adelaide, SA Australia; 10 Well Child Laboratory, St Thomas’ Hospital, Evelina London Children’s Hospital, London, UK; 11 Division of Endocrinology, Hospital for Sick Children, Toronto, ON Canada; 12 Department of Endocrinology and Diabetes, Perth Children’s Hospital, Perth, WA Australia; 13 The Kids Research Institute Australia, Nedlands, WA Australia; 14 Translational and Clinical Research Institute, Newcastle University, Newcastle, UK; 15 Oxford Centre for Diabetes, Endocrinology and Metabolism, Oxford, UK; 16 Department of Paediatrics, University of Cambridge, Cambridge, UK; 17 Department of Paediatric Diabetes and Endocrinology, Cambridge University Hospitals NHS Foundation Trust, Cambridge, UK

**Keywords:** AdDIT, Adolescents, Diabetes, type 1, Cardiovascular, Complications, Vascular diseases, Diseases, Endocrinology

## Abstract

Adolescence represents a pivotal period for the development of early diabetes-related vascular complications, primarily driven by chronic hyperglycemia and associated metabolic disturbances. Metabolomics provides a promising tool to identify biomarkers reflecting these early changes. The present study compared metabolomic profiles between adolescents with and without type 1 diabetes and assessed the impact of glycemia and short-term treatment with statins and/or ACE inhibitors on metabolite trajectories. Metabolomic data were obtained via the Nightingale platform from 341 adolescents with type 1 diabetes from the AdDIT study and 81 age- and sex-matched peers without diabetes. Two hundred and forty-nine metabolites, with 45 prioritized due to well-established links with cardiovascular disease- including lipid subfractions, amino acids, ketone bodies, and markers of renal function and inflammation—were measured at a mean age of 14.0 ± 1.8 years and re-assessed after 3.5 years in those with diabetes. At baseline, significant differences were observed in glucose, ketone bodies, amino acids, and lipid classes. Over time, adolescents with diabetes showed increases in lipid subfractions, acetoacetate, and GlycA, with HbA1c positively associated with atherogenic lipidome components. Findings from the trial arm provided strong evidence that statins attenuated rises in LDL-cholesterol (1.90 [1.79, 2.01] vs. 2.38 [2.28, 2.49] mmol/l), and weaker evidence suggestive of a small effect of ACE inhibitors in preserving HDL-cholesterol (1.50 [1.45, 1.54] vs. 1.39 [1.34, 1.43] mmol/l). These findings suggest widespread glycemia-related metabolomic perturbations in adolescents with type 1 diabetes, partially mitigated by statins and ACE inhibitors, supporting their cardioprotective role.

*Clinical registration*: ClinicalTrials.gov ID NCT01581476 (13/04/2012) https://clinicaltrials.gov/study/NCT01581476.

**Supplementary Information:**

The online version contains supplementary material available at 10.1038/s41598-026-50044-w.

## Introduction

Adolescence represents a crucial period for the development of early stages of vascular complications in people with type 1 diabetes^1^, which are driven by hyperglycemia and other risk factors, including overweight/obesity, insulin resistance, dyslipidemia, high blood pressure and lifestyle factors, such as smoking^2–4^. Perturbations in numerous metabolic pathways and biochemical processes may lay the groundwork for vascular complications^5^. Thus, early detection and prevention of complications should start during adolescence, and this could be supported by biomarkers reflecting early metabolic and vascular changes^4^.

Metabolomics, which allows the identification and quantification of metabolites in biological fluids and tissues, has been applied in many studies to identify potential biomarkers to predict disease onset or prognosis, as well as to monitor the effect of pharmacological interventions^6,7^. Previous studies have reported metabolic alterations in carbohydrates, amino acids, ketone bodies, nucleotides, vitamin D metabolites, steroids, lipids and free fatty acids (FFAs), primarily in adults with type 1 diabetes. Of note, interventions improving glycemia restored some of these metabolic perturbations^8–11^. Scant data are available on metabolomic changes in adolescents with type 1 diabetes, and how they are related to glycemic variations and affected by therapeutic interventions.

The Adolescent Type 1 Diabetes cardio-renal Intervention Trial (AdDIT) demonstrated beneficial effects of statins on lipid levels and of angiotensin converting enzyme (ACE) inhibitors on albuminuria and endothelial dysfunction^12,13^. However, the effect of these interventions on metabolic factors implicated in the increased risk of vascular complications remains unclear.

The aim of the present study was to characterize the relationship of glycemia and the impact of early short-term treatment with statins and ACE inhibitors on metabolomic biomarkers encompassing lipid subfractions, amino acid, ketone bodies and glycolysis-related metabolites and markers of renal function and inflammation in adolescents with type 1 diabetes.

## Results

### Baseline characteristics

Clinical and biochemical characteristics of the study population are summarized in Table 1.

**Table 1:** Clinical and biochemical characteristics of the study population.

	Group with type 1 diabetes					Group without type 1 diabetes
	AdDIT RCT*				AdDIT Observational	Baseline
	ACE inhibitors	Placebo	Statins	Placebo		
N	74	67	74	67	200	81
Sex (% female)	45	39	39	45	46	48
Age at Diagnosis (yr)	8.9 ± 3.5	8.9 ± 3.4	8.7 ± 3.7	9.1 ± 3.2	6.6 ± 3.3	-
T1D Duration (yr)	5.0 ± 3.4	4.9 ± 3.1	5.3 ± 3.2	4.7 ± 3.3	7.3 ± 3.3	-
Study Follow-Up Duration (yr)	3.5 ± 0.7	3.4 ± 0.6	3.4 ± 0.6	3.5 ± 0.7	3.9 ± 0.4	-
Age (yr)						
Baseline	13.9 ± 1.6	13.8 ± 1.7	13.9 ± 1.8	13.8 ± 1.5	13.9 ± 1.6	14.2 ± 2.3
Follow-Up	17.5 ± 1.8	17.2 ± 1.8	17.4 ± 1.9	17.3 ± 1.6	17.8 ± 1.7	-
BMI (kg/m^2^)						
Baseline	20.0 (18.4–23.0)	19.7 (17.5–23.0)	19.7 (17.9–23.0)	20.2 (18.0–22.9)	21.2 (19.0–23.7)	19.9 (18.4–22.2)
Follow-Up	22.5 (21.0–26.0)	22.0 (19.7–25.8)	21.9 (20.8–24.5)	23.0 (20.3–26.5)	23.7 (21.4–26.6)	-
HbA1c (%)						
Baseline	8.1 ± 1.2	8.4 ± 1.3	8.1 ± 1.2	8.4 ± 1.2	8.4 ± 1.2	-
Follow-Up	8.6 ± 1.6	8.7 ± 1.8	8.6 ± 1.6	8.7 ± 1.8	8.6 ± 1.5	
HbA1c (mmol/mol)						
Baseline	64.7 ± 12.7	68.0 ± 14.2	64.8 ± 13.3	67.9 ± 13.6	68.7 ± 12.8	-
Follow-Up	70.4 ± 17.6	72.9 ± 19.7	71.0 ± 17.6	71.3 ± 19.7	70.9 ± 16.8	
LDL-c (mmol/L)						
Baseline	2.35 ± 0.66	2.35 ± 0.66	2.28 ± 0.72	2.32 ± 0.61	2.57 ± 0.73	2.20 ± 0.60
Follow-Up	2.27 ± 0.96	2.11 ± 0.84	1.90 ± 0.91	2.48 ± 0.80	2.3 ± 0.70	-
HDL-c (mmol/L)						
Baseline	1.62 ± 0.41	1.55 ± 0.38	1.63 ± 0.45	1.55 ± 0.33	1.6 ± 0.40	1.47 ± 0.31
Follow-Up	1.54 ± 0.41	1.40 ± 0.35	1.47 ± 0.38	1.48 ± 0.40	1.46 ± 0.39	-
Triglycerides (mmol/L)						
Baseline	0.7 (0.6–1.1)	0.8 (0.6–1.1)	0.8 (0.6–1.1)	0.8(0.6–1.2)	0.8 (0.6–1.1)	0.7 (0.6–1.0)
Follow-Up	0.9 (0.7–1.2)	0.9 (0.7–1.3)	0.8 (0.7–1.1)	0.9 (0.7–1.3)	0.9 (0.7–1.2)	-
hsCRP (mg/L)						
Baseline	0.4 (0.2–1.1)	0.4 (0.2 -1.0)	0.4 (0.2–1.1)	0.5 (0.2–1.0)	0.5 (0.2–1.2)	0.5 (0.2–1.0)
Follow-Up	0.7 (0.3–1.8)	0.5 (0.3–2.0)	0.6 (0.3–1.4)	0.8 (0.3–2.5)	0.7 (0.3–2.3)	-

Data are mean ± SD or median (interquartile range).

AdDIT: Adolescent Type 1 Diabetes cardio-renal Intervention Trial; BMI: body mass index; T1D: type 1 diabetes; HbA1c: glycated hemoglobin; HDL-c: high-density lipoprotein–cholesterol; hsCRP: high-sensitivity C-reactive protein; LDL-c: low-density lipoprotein-cholesterol; RCT: randomized controlled trial.

*AdDIT RCT population: 34 participants were randomized to receive ACE inhibitors alone, 34 to receive statins alone, 40 to receive both ACE inhibitors and statins, and 33 to receive placebo alone. Due to the 2 × 2 factorial nature of the trial, this resulted in an ACE inhibitor arm consisting of 74 participants on ACE inhibitors and 67 not, and a statin arm with 74 participants assigned to statin therapy and 67 not.

A total of 422 adolescents were enrolled in the study, including 341 adolescents with type 1 diabetes (mean age (± SD) 13.9 ± 1.6, females 44%), who were re-assessed after 3.5 years of follow up; and 81 peers without type 1 diabetes (age: 14.2 ± 2.3, females 48%), with available data only at the baseline visit. The two groups were comparable for age, sex and BMI. There were no significant differences in low-density lipoprotein (LDL)-cholesterol, triglycerides or high-sensitivity C-reactive protein (hsCRP) between the two groups, whereas levels of high-density lipoprotein (HDL)-cholesterol were slightly higher in the group with type 1 diabetes. Within the type 1 diabetes group, there was little evidence for differences between adolescents in the RCT and Observational arms, apart from a longer diabetes duration in those in the Observational group.

### Metabolites at baseline

**Fig. 1:**
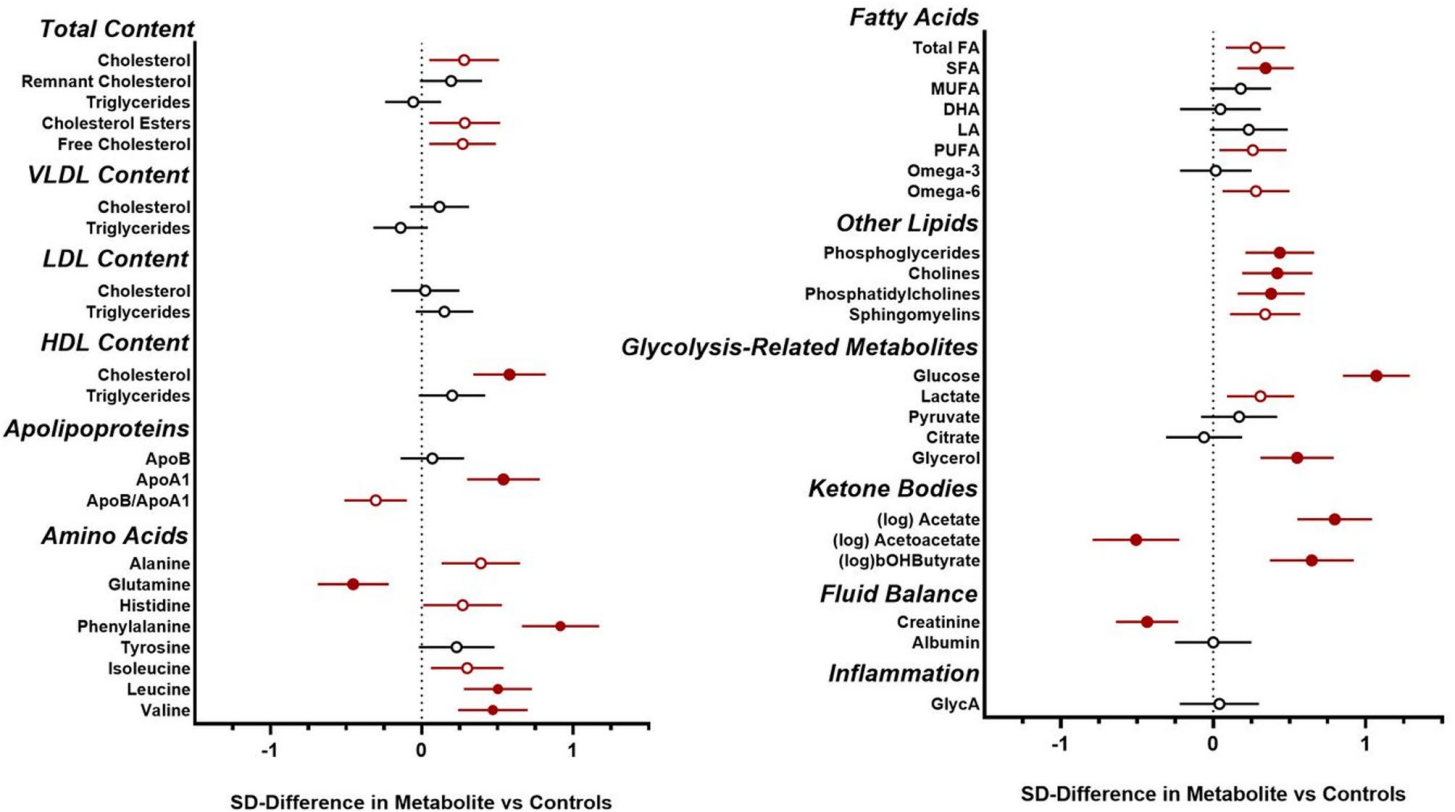
Comparison of early adolescent metabolomic profiles in T1D and controls. Results show a z-score mean difference (+/- 95% CIs) for each outcome in individuals with T1D relative to controls. Red hollow symbols indicate results reaching significance level of *p* < 0.05, while solid symbols represent results remaining significant after Benjamini-Hochberg corrections for multiple testing.

At baseline, participants with type 1 diabetes had higher levels of glucose, glycerol and β-hydroxybutyrate (BHB) compared to those without diabetes (Fig. 1). Those with type 1 diabetes also had higher levels of phenylalanine, leucine and valine, phosphoglycerides, cholines, phosphatidylcholines, HDL particles cholesterol content, Apolipoprotein A1 (ApoA1) and saturated fatty acids (SFAs), whereas creatinine and acetoacetate levels were lower (Fig. 1).

### Metabolite changes during follow up

During follow-up, participants with type 1 diabetes in the Observational arm showed a consistent, upward trend in all measured lipid components, apart from cholesterol content in HDL particles, which declined. Levels of fatty acids (FAs), phosphoglycerides, cholines, phosphatidylcholines, acetoacetate, glycoprotein acetyls (GlycA) and creatinine also increased. In contrast, most amino acids and citrate levels declined (Table 2).

**Table 2:** Percent change in metabolite levels over 3.5 years of follow-up in untreated individuals with type 1 diabetes.

	% Change	Lower 95%CI	Upper 95%CI	*p*-value
Total Content				
Cholesterol	7	5	10	**< 0.001**
Remnant cholesterol	12	8	15	**< 0.001**
Triglycerides	28	23	34	**< 0.001**
Cholesterol esters	7	5	10	**< 0.001**
Free cholesterol	8	6	11	**< 0.001**
VLDL Content				
Cholesterol	16	11	20	**< 0.001**
Triglycerides	38	31	44	**< 0.001**
LDL Content				
Cholesterol	14	11	17	**< 0.001**
Triglycerides	14	10	18	**< 0.001**
HDL Content				
Cholesterol	− 3	− 6	− 1	0.012
Triglycerides	14	9	19	**< 0.001**
Apolipoproteins				
ApoB	11	8	14	**< 0.001**
ApoA1	0	− 2	2	0.785
ApoB / ApoA1	11	9	14	**< 0.001**
Amino Acids				
Alanine	− 4	− 7	− 1	0.004
Glutamine	− 2	− 4	1	0.181
Histidine	− 8	− 11	− 6	**< 0.001**
Phenylalanine	− 23	− 26	− 20	**< 0.001**
Tyrosine	− 10	− 14	− 7	**< 0.001**
Isoleucine	− 5	− 9	0	0.04
Leucine	− 11	− 15	− 7	**< 0.001**
Valine	− 4	− 7	− 1	0.003
Fatty Acids				
Total FA	9	6	12	**< 0.001**
SFA	11	7	14	**< 0.001**
MUFA	13	9	17	**< 0.001**
DHA	4	0	8	0.064
LA	8	5	11	**< 0.001**
PUFA	6	4	8	**< 0.001**
Omega-3	8	3	13	0.003
Omega-6	6	3	8	**< 0.001**
Other Lipids				
Phosphoglygerides	6	3	9	**< 0.001**
Cholines	5	3	7	**< 0.001**
Phosphatidylcholines	8	5	10	**< 0.001**
Sphingomyelins	4	1	6	0.002
Glycolysis-related Metabolites				
Glucose	1	− 7	9	0.745
Lactate	2	− 4	7	0.509
Pyruvate	9	3	16	0.009
Citrate	− 22	− 28	− 16	**< 0.001**
Glycerol	− 9	− 14	− 3	0.001
Ketone Bodies and SCFAs				
(log) Acetate	− 10	− 20	0	0.035
(log) Acetoacetate	224	204	243	**< 0.001**
(log) β-hydroxybutyrate	− 8	− 24	9	0.337
Renal				
Creatinine	20	18	22	**< 0.001**
Albumin	0	− 2	1	0.731
Inflammation				
GlycA	4	2	7	**< 0.001**

Bold p-values represent results remaining significant after Benjamini-Hochberg corrections for multiple testing.AA: amino acid; ApoA1: Apolipoprotein A1; ApoB: Apolipoprotein B; BHB: β-hydroxybutyrate; DHA: Docosahexaenoic acid; FA: fatty acid; FFA: free fatty acid; HbA1c: glycated hemoglobin, GlycA: glycoprotein acetyls HDL: High-Density Lipoprotein; hsCRP: high-sensitivity C-Reactive Protein; KB: Ketone bodies; LA: Lactic acid; LDL: Low-Density Lipoprotein; log: logarithm; MUFA: Monounsaturated Fatty Acid; PUFA: Polyunsaturated Fatty Acid; SCFA short chain fatty acid; SFA: saturated fatty acid.

Increases in glycated hemoglobin (HbA1c) during follow up were positively associated with all lipid components (apart from cholesterol content in HDL particles), apolipoproteins, FAs, phosphoglycerides, cholines, phosphatidylcholines, sphingomyelins, GlycA. In contrast, negative associations were found between glutamine, histidine and citrate levels and HbA1c. (Table 3)

**Table 3:** Associations between HbA1c at baseline and during follow up and metabolite levels in late adolescence (17.5 years).

	HbA1c			
	Baseline	*p*-value	Increase over time	*p*-value
Total Content				
Cholesterol	0.05 (− 0.02–0.11)	0.140	**0.10 (0.05–0.15)**	**< 0.001**
Remnant cholesterol	0.02 (− 0.04–0.09)	0.525	**0.11 (0.06–0.16)**	**< 0.001**
Triglycerides	0.01 (− 0.07–0.09)	0.775	**0.16 (0.10–0.22)**	**< 0.001**
Cholesterol esters	0.05 (− 0.01–0.12)	0.116	**0.09 (0.04–0.14)**	**< 0.001**
Free cholesterol	0.04 (− 0.03–0.10)	0.234	**0.11 (0.06–0.16)**	**< 0.001**
VLDL Content				
Cholesterol	0.01 (− 0.06–0.08)	0.746	**0.13 (0.08–0.18)**	**< 0.001**
Triglycerides	0.01 (− 0.078- 0.09)	0.895	**0.15 (0.09–0.22)**	**< 0.001**
LDL Content				
Cholesterol	0.06 (0.01–0.12)	0.102	**0.10 (0.05–0.15)**	**< 0.001**
Triglycerides	0.05 (− 0.03–0.13)	0.189	**0.17 (0.12–0.23)**	**< 0.001**
HDL Content				
Cholesterol	0.03 (− 0.03–0.09)	0.372	0.03 (− 0.02–0.08)	0.228
Triglycerides	0.01 (− 0.07–0.08)	0.816	**0.13 (0.07–0.18)**	**< 0.001**
Apolipoproteins				
ApoB	0.03 (− 0.03–0.09)	0.365	**0.11 (0.07–0.16)**	**< 0.001**
ApoA1	0.04 (− 0.02–0.11)	0.187	**0.06 (0.01–0.11)**	**0.017**
ApoB / ApoA1	− 0.01 (− 0.07–0.04)	0.625	**0.08 (0.03–0.12)**	**0.001**
Amino Acids				
Alanine	0.00 (− 0.07–0.06)	0.942	0.03 (− 0.02–0.08)	0.291
Glutamine	**− 0.07 (− 0.14 to − 0.01)**	**0.027**	**− 0.11 (− 0.16 to − 0.06)**	**< 0.001**
Histidine	− 0.01 (− 0.07–0.06)	0.814	**− 0.10 (− 0.15 to − 0.04)**	**< 0.001**
Phenylalanine	0.04 (− 0.04–0.12)	0.368	− 0.01 (− 0.07–0.06)	0.873
Tyrosine	0.02 (− 0.06–0.10)	0.628	− 0.03 (− 0.09–0.03)	0.357
Isoleucine	0.04 (− 0.04–0.11)	0.311	0.02 (− 0.03–0.08)	0.450
Leucine	0.05 (− 0.02–0.13)	0.176	0.03 (− 0.03–0.09)	0.271
Valine	0.05 (− 0.02–0.12)	0.188	**0.06 (0.01–0.12)**	**0.028**
Fatty Acids				
Total FA	0.05 (− 0.03–0.13)	0.205	**0.16 (0.10–0.22)**	**< 0.001**
SFA	0.05 (− 0.03–0.13)	0.205	**0.16 (0.10–0.21)**	**< 0.001**
MUFA	0.05 (− 0.03–0.13)	0.247	**0.18 (0.12–0.24)**	**< 0.001**
DHA	**0.09 (0.02–0.16)**	**0.016**	**0.07 (0.01–0.13)**	**0.014**
LA	0.04 (− 0.03–0.11)	0.270	**0.12 (0.07–0.17)**	**< 0.001**
PUFA	0.04 (− 0.03–0.11)	0.224	**0.13 (0.07–0.18)**	**< 0.001**
Omega-3	0.01 (− 0.05–0.08)	0.712	**0.11 (0.05–0.16)**	**< 0.001**
Omega-6	0.05 (− 0.02–0.12)	0.182	**0.13 (0.07–0.18)**	**< 0.001**
Other Lipids				
Phosphoglygerides	0.05 (− 0.02–0.12)	0.131	**0.11 (0.06–0.17)**	**< 0.001**
Cholines	0.05 (− 0.02–0.12)	0.131	**0.11 (0.05–0.16)**	**< 0.001**
Phosphatidylcholines	0.05 (− 0.02–0.12)	0.172	**0.11 (0.06–0.16)**	**< 0.001**
Sphyngomyelins	0.06 (0.00–0.13)	0.061	**0.08 (0.03–0.13)**	**0.003**
Glycolysis-related Metabolites				
Glucose	0.06 (− 0.01–0.14)	0.111	0.06 (0.00–0.11)	0.052
Lactate	0.04 (− 0.04–0.12)	0.335	**0.06 (0.00–0.12)**	**0.049**
Pyruvate	0.05 (− 0.02–0.12)	0.176	0.05 (0.00–0.11)	0.073
Citrate	− 0.00 (− 0.08–0.07)	0.949	**− 0.09 (− 0.15 to − 0.03)**	**0.005**
Glycerol	0.07 (− 0.00–0.15)	0.064	**0.11 (0.05–0.17)**	**< 0.001**
Ketone Bodies and SCFAs				
(log) Acetate	− 0.04 (− 0.11–0.03)	0.261	0.01 (− 0.05–0.07)	0.743
(log) Acetoacetate	**0.17 (0.07–0.28)**	**0.001**	0.06 (− 0.02–0.14)	0.171
(log) hydroxybutyrate	**0.14 (0.05–0.23)**	**0.002**	0.04 (− 0.03–0.11)	0.209
Renal				
Creatinine	− 0.05 (− 0.12–0.01)	0.102	0.01 (− 0.04–0.06)	0.775
Albumin	− 0.02 (− 0.10–0.05)	0.575	− 0.03 (− 0.10–0.03)	0.268
Inflammation				
GlycA	0.05 (− 0.02–0.13)	0.148	**0.11 (0.05–0.17)**	**< 0.001**

Baseline exposures adjusted for age, sex, duration of disease, baseline metabolite level, and country. Increase over time exposure derived from residuals of final metabolite value regressed against baseline value.

AA: amino acid; ApoA1: Apolipoprotein A1; ApoB: Apolipoprotein B; BHB: β-hydroxybutyrate; DHA: Docosahexaenoic acid; FA: fatty acid; FFA: free fatty acid; HbA1c: glycated hemoglobin, GlycA: glycoprotein acetyls HDL: High-Density Lipoprotein; hsCRP: high-sensitivity C-Reactive Protein; KB: Ketone bodies; LA: Lactic acid; LDL: Low-Density Lipoprotein; log: logarithm; MUFA: Monounsaturated Fatty Acid; PUFA: Polyunsaturated Fatty Acid; SCFA: short chain fatty acid; SFA: saturated fatty acid.

### Effect of statins and ACE-inhibitors on metabolites changes over time

**Fig. 2:**
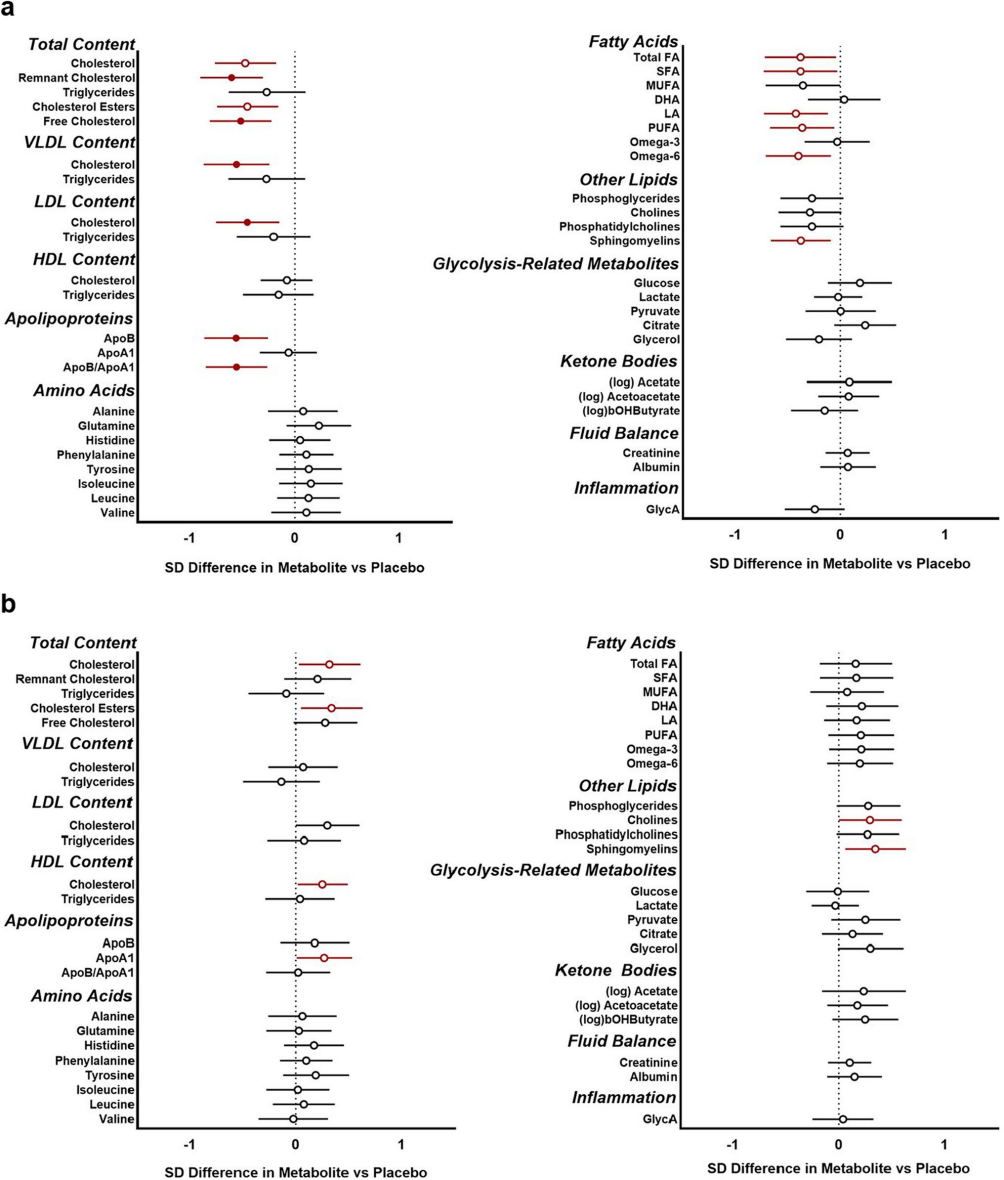
Effect of statins (**a**) and ACE inhibitors (**b**) on metabolomic profile in T1D. Forest plots show mean z-score difference (+/- 95% CIs) at follow up in individuals taking ACE inhibitors / statins vs those taking placebo. Red hollow symbols indicate results reaching significance level of p < 0.05, while solid symbols represent results remaining significant after Benjamini-Hochberg corrections for multiple testing.

Changes in metabolites in participants treated with statin or ACE inhibitor vs. those in the placebo group are shown in Fig. 2a and b, respectively.

Statin treatment was associated with lower levels of free cholesterol, remnant cholesterol and the cholesterol content of LDL-cholesterol and very-low density (VLDL)-cholesterol particles at trial end. Moreover, statin use was associated with lower levels of most FAs, sphingomyelins and ApoB.

**Fig. 3:**
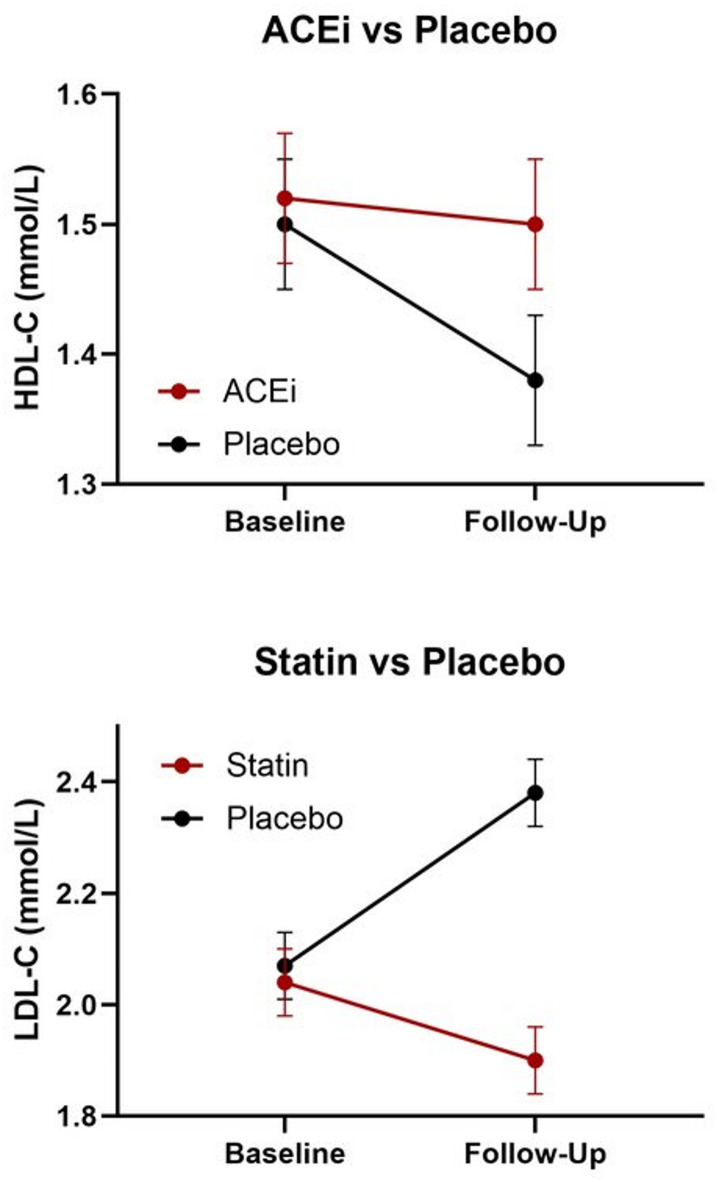
Changes over time in LDL- and HDL-cholesterol in participants treated with statin vs. placebo ACE inhibitors vs. placebo, respectively. Data are mean (+/- 95% CIs).

Participants on ACE inhibitors showed higher HDL particle cholesterol content, ApoA1, total cholesterol content and its esters, choline, and sphingomyelin compared to placebo recipients, although these differences did not achieve statistical significance. The effects of statins on LDL-cholesterol and ACE inhibitors on HDL-cholesterol are illustrated in Fig. 3. Specifically, the reduced LDL-cholesterol concentrations documented in statin-treated participants and the trend toward higher HDL-cholesterol levels observed in those treated with ACE inhibitors can be attributed to the favorable effects of statins and ACE inhibitors in preventing LDL-cholesterol increase and HDL-cholesterol reduction, respectively.

### Supplementary Information

The results for the remaining 204 metabolites, not included in the primary analyses, are reported in the supplementary material (Supplementary Fig. 1–3 and Table 1, and 2).

## Discussion

This study provides evidence of widespread metabolomic perturbations—primarily in lipid subfractions—in adolescents with type 1 diabetes compared to their peers without diabetes. Our findings indicate that atherogenic metabolic profiles worsened over time during the transition from early to late adolescence, a deterioration that was closely linked to a parallel rise in HbA1c. Notably, we observed that treatment with statins, and to a lesser extent ACE inhibitors, led to improvements in lipid fractions, or at least prevented deterioration over time.

Of note, treatment with statins in our study population appeared to mitigate the deterioration of metabolomic profiles over time, demonstrating a clear effect in preventing increases in atherogenic lipid subfractions compared to placebo. Findings also suggested that ACE inhibitors may attenuate the decline in HDL-cholesterol, although these findings did not reach FDR-corrected levels of significance and should therefore be treated with caution until replicated in additional or larger cohorts. The lipid-lowering effects associated with statin treatment, which is consistent with previous findings^14^, could have beneficial effects on vascular health. This may be achieved by shifting the trajectories of lipid components toward trends more closely resembling those seen in adolescents without diabetes, who physiologically experience a decline in lipid levels during puberty^15,16^. Conversely, it can be postulated that the impact of ACE inhibitors on HDL-cholesterol may contribute to the preservation of renal function^17^, and/or endothelial function^13^.

Levels of several lipid subfractions, including total cholesterol, free cholesterol and esters, FAs, phosphoglycerides, cholines, phosphatidylcholines and sphingomyelins were higher in adolescents with type 1 diabetes than their peers. Those with type 1 diabetes also had higher levels of HDL cholesterol content, ApoA1 and a reduced ApoB/A1 ratio, whereas creatinine levels were lower, and this could reflect renal hyperfiltration^18^ and/or the lower muscle mass observed in individuals with type 1 diabetes^19,20^. On the other hand, the observed increase in creatinine levels at the end of the follow-up period in individuals with type 1 diabetes is likely related to age-related increases during adolescence^21^.

Previous studies have reported that adolescents with type 1 diabetes have higher levels of HDL-cholesterol and ApoA1 in comparison to those without diabetes^22–24^. Although these findings might suggest a better cardiovascular profile, the expected cardioprotective effects of these lipids could be reduced due to alterations in the functions and structure of HDL particles induced by chronic hyperglycemia^25^. Increased ApoA1 levels may represent a compensatory mechanism aimed at mitigating the functional deficit of HDL particles^26^.

As expected and in agreement with longstanding evidence, chronic hyperglycemia was associated with higher circulating levels of BHB. In individuals with type 1 diabetes, serum levels of BHB are more elevated than acetoacetate, and this was confirmed in our study population^27^. This phenomenon is attributable to reduced capacity to metabolize BHB and decreased peripheral conversion of BHB into acetoacetate^28^. Furthermore, reduced ketone bodies clearance^29^ and/or impaired liver function can also contribute to this imbalance^30^. Increased serum levels of ketone bodies can induce oxidative stress, particularly in cardiomyocytes, red blood cells and endothelial cells, as well as systemic inflammation^31,32^, and in this way contribute to the pathogenesis of vascular complications^33^. Of note, our results revealed lower baseline acetoacetate levels in individuals with type 1 diabetes, with levels increasing over time. This contrasts with the higher baseline levels of BHB, and the precise mechanisms underlying this finding warrant further investigation.

The present study also showed that individuals with type 1 diabetes had increased levels of amino acids, which may be due to increased protein catabolism associated with insulin deficiency^34^.

Previous studies, including mainly adult populations, highlighted several metabolic perturbations in individuals with type 1 diabetes. Balderas and colleagues^8^ compared the metabolomic profile of children with and without type 1 diabetes and identified higher levels of FFAs, sphingoid and lysophospholipids in the latter group, in line with our study. Additionally, lower citrate levels were observed, a finding that is consistent with the longitudinal results in our Observational cohort. Decreased citrate levels could potentially be related to insulin deficiency, which leads to an increase in gluconeogenesis and conversion of pyruvate into oxaloacetate rather than acetyl-CoA^35^.

Bervoets et al.^10^ compared metabolomic profiles of children and adolescents with type 1 diabetes with those of peers without diabetes. In line with our findings, individuals with diabetes had higher levels of glucose and ketone bodies. Conversely, they displayed lower levels of phospholipids, colinated phospholipids and triglycerides. In contrast to our findings, Sobczak and colleagues reported reduced levels of FFAs in adults with type 1 diabetes and a negative correlation between HbA1c and FFAs^11^. Differences between studies could be related to the demographic and clinical characteristics of the study population and their glycemic profile.

The present study also highlighted that atherogenic metabolic profiles worsened over time and these changes were closely linked to a parallel trend in HbA1c. In addition to the clear associations between HbA1c and lipid subfractions, a positive correlation was observed between GlycA and HbA1c over time. GlycA is a marker of systemic inflammation that simultaneously reflects increased glycan complexity and elevated levels of circulating acute-phase proteins. It has been described as a more sensitive measure than hsCRP for detecting early cardiovascular risk^36–38^.

However, a limitation of the present study is the lack of longitudinal data for the group of adolescents without diabetes, which prevents the full exclusion of potential physiological age-related changes as contributors to the observed metabolic patterns over time, although analyses were adjusted for age. In addition, in our study we did not collect any information on physical activity or diet, which are two potential confounding factors when assessing metabolic markers.

## Methods

### Study population

The study population included 341 adolescents with type 1 diabetes and 81 peers without diabetes. Participants with type 1 diabetes were a subgroup of the larger cohort enrolled in the AdDIT studies (Observational and Trial). The overall AdDIT design has been previously reported along with the protocol and statistical plan^12,39,40^. Briefly, between 2009 and 2013, 443 adolescents (aged 10–16 years) with a urinary ACR in the upper tertile of the normal range (high-ACR group) were randomized to receive a statin (Atorvastatin) or matching placebo, an ACE-inhibitor (Quinapril) or matching placebo, or a combination of the two drugs in a 2 × 2 factorial design over a treatment period of 2–4 years^12,39,40^.

The AdDIT trial and related protocol and statistical analysis plan were previously published^12^ https://www.nejm.org/doi/suppl/10.1056/NEJMoa1703518/suppl_file/nejmoa1703518_protocol.pdf). During the same period, 404 adolescents with an ACR in the middle or lower tertile (low-ACR group) were recruited into the parallel AdDIT Observational study. Both groups underwent similar baseline and follow-up assessments based on a standardized protocol^39,40^.

Out of 341 adolescents with type 1 diabetes included in the present study, 141 were from the AdDIT trial, whereas the remaining 200 individuals were part of the AdDIT Observational study^12,40^. Within the trial itself, 34 participants were randomized to receive ACE inhibitors alone, 34 to receive statins alone, 40 to receive both ACE inhibitors and statins, and 33 to receive placebo alone. Due to the 2 × 2 factorial nature of the trial, this resulted in an ACE inhibitor arm consisting of 74 participants on ACE inhibitors and 67 not, and a statin arm with 74 participants assigned to statin therapy and 67 not. Participants had metabolomics analysis carried out on samples collected at baseline and at the last follow-up visit, after a median follow-up of 3.5 years.

The control group included 81 age- and sex-matched youths without type 1 diabetes, who were recruited as a control group at AdDIT Australian and Canadian clinical sites during the same timeframe of the AdDIT studies. Samples from controls were collected at baseline only.

The study was sponsored by the University of Cambridge and Cambridge University Hospitals NHS Foundation Trust. The study protocol adhered to the Declaration of Helsinki and was approved by South West research ethics committee (REC ref no 08/H0206/4) and then by other local research ethics committees. Parents of participants provided appropriate written informed consent, and study participants were asked to provide their assent until they reached an age at which they could consent to study follow-up. All data collection and analyses were performed in accordance with relevant guidelines and regulations.

## Methods

All data collection and analyses were performed in accordance with relevant guidelines and regulations.

### Study assessments

At baseline and end of the study, standardized assessments were performed, including measurements of height (cm), weight (kg), and arterial blood pressure (mmHg). Non-fasting blood samples were collected for local HbA1c measurements (using DCCT aligned assays) and for centralized measurements of renal and cardiovascular markers. Additional samples were collected and stored at − 80 °C and subsequently used to measure biomarkers for the present study.

### Metabolites

Metabolite quantification was performed using a proprietary NMR metabolite profiling service (Nightingale Health, Helsinki, Finland). Plasma samples were analyzed using 500 MHz NMR spectrometers (Bruker AVANCE IIIHD). Two NMR spectra were recorded for each plasma sample. The first is a presaturated proton spectrum, which features resonances arising mainly from proteins, lipids and lipoprotein particles. The second is a Carr-Purcell-Meiboom-Gill (CPMG) T2-relaxation-filtered spectrum, which enhances the detection of low-molecular-weight metabolites. The metabolites and lipoprotein fractions were quantified using Nightingale Health’s proprietary software (quantification library 2020), which simultaneously quantifies 249 metabolic measures per EDTA plasma sample, comprising 168 absolute and 81 ratio measures.

The primary reported analyses for the NMR-based metabolite profiling are restricted to 45 metabolites out of 249 available in all participants. These metabolites were chosen based on existing literature indicating their associations with the outcomes of interest. They included lipid subfractions, amino acids, ketone bodies, glycolysis-related metabolites and markers of renal function and inflammation. The absolute concentrations of metabolites were prioritized over NMR-derived ratio measures. Details of metabolites not included in the main analyses are reported as Supplementary material.

### Statistical analysis

Descriptive data are summarized as mean ± SD or median (interquartile range) unless otherwise specified. Normality was assessed via visual inspection of histograms and metabolites with highly skewed distributions log-transformed before analysis. Between group comparisons were performed by unpaired t-tests for continuous variables and by χ2 or Fisher’s exact test for categorical variables. Mixed linear models were used to assess differences in metabolite levels between individuals with and without type 1 diabetes, and to assess differences between the active treated and placebo groups in the randomized clinical trial. For comparisons between individuals with and without type 1 diabetes, models were adjusted for age, sex, and BMI, with country (UK, Australia, or Canada) also included as a random effect. For the assessment of drug effects in the clinical trial, models were additionally adjusted for duration of disease, HbA1c, and initial metabolite level. Change in metabolite levels over time in individuals with type 1 diabetes (observational cohort and placebo arm of RCT combined) were assessed using mixed models with repeated measures adjusted for age, sex, BMI, initial metabolite level, and time in trial. All outcomes were log-transformed prior to inclusion in models and then back-transformed prior to reporting, with values therefore representing a percentage change in geometric means over time. Finally, associations between HbA1c and metabolite levels were also assessed using mixed linear models adjusted for age, sex, duration of disease, and baseline metabolite level, with country again fitted as a random effect. Increase over time exposures were derived from residuals of final metabolite value regressed against baseline value. All analyses were carried out using Stata v18 (College Station, TX: StataCorp LLC.). The interpretation of the results was primarily based on estimates and 95% CIs, rather than statistical significance levels relative to arbitrary FDR-adjusted p-values. The latter – calculated using the Benjamini-Hochberg method - are still provided throughout, however, for reference.

## Conclusions

Youth with type 1 diabetes exhibit significant disruptions in their metabolomic profiles during early adolescence. Notably, these disturbances, especially those affecting the lipidome, tend to worsen as HbA1c levels increase over time. The use of statins, and to a lesser extent, ACE inhibitors, has the potential to ameliorate some of these disruptions, which could contribute to the prevention of vascular complications.

The impact of statins and ACE inhibitors on both atherogenic and protective components of the lipidome lays the groundwork for further investigation into the clinical implications of these findings in adolescents with type 1 diabetes.

## Supplementary Information

Below is the link to the electronic supplementary material.

Supplementary Material 1

## Data Availability

The data that support the findings of this study are available from the corresponding author upon reasonable request. All statistical scripts for analyses conducted in this paper can be found in an open access resource at https://github.com/scottchiesa/AdDIT_Metabolomics.

## Abbreviations

ACE: Angiotensin converting enzyme
AdDIT: Adolescent Type 1 Diabetes cardio-renal Intervention tTrial
ACR: Albumin-to-creatinine ratio
AA: Amino acid
ApoA1: Apolipoprotein A1
ApoB: Apolipoprotein B
BHB: β-hydroxybutyrate
CI: Confidence Interval
DHA: Docosahexaenoic acid
FDR: False discovery rate
FA: Fatty acid
FFA: Free fatty acid
HbA1c: Glycated hemoglobin
GlycA: Glycoprotein acetyls
HDL: High density lipoprotein
hsCRP: High sensitivity C-reactive protein
KB: Ketone bodies
LA: Lactic acid
log: logarithm
LDL: Low density lipoprotein
MUFA: Monounsaturated fatty acid
PUFA: Polyunsaturated fatty acid
RCT: Randomized controlled trial
SCFA: Short chain fatty acid
SFA: Saturated fatty acid
VLDL: Very low density lipoprotein

## References

[1] Chiesa, S. T. & Marcovecchio, M. L. Preventing cardiovascular complications in type 1 diabetes: The need for a lifetime approach. *Front. Pediatr.* **9**, 696499. 10.3389/fped.2021.696499 (2021). 34178905 10.3389/fped.2021.696499 PMC8219852

[2] Batch, J. A., Baxter, R. C. & Werther, G. Abnormal regulation of insulin-like growth factor binding proteins in adolescents with insulin-dependent diabetes. *J. Clin. Endocrinol. Metab.* **73** (5), 964–968. 10.1210/jcem-73-5-964 (1991). 1719017 10.1210/jcem-73-5-964

[3] Clements, M. A. et al. Hemoglobin A1c (HbA1c) changes over time among adolescent and young adult participants in the T1D exchange clinic registry. *Pediatr. Diabetes*. **17** (5), 327–336. 10.1111/pedi.12295 (2016). 26153338 10.1111/pedi.12295

[4] Bjornstad, P. et al. ISPAD Clinical Practice Consensus Guidelines 2022: Microvascular and macrovascular complications in children and adolescents with diabetes. *Pediatr. Diabetes*. **23** (8), 1432–1450. 10.1111/pedi.13444 (2022). 36537531 10.1111/pedi.13444

[5] Frohnert, B. I. & Rewers, M. J. Metabolomics in childhood diabetes. *Pediatr. Diabetes*. **17** (1), 3–14. 10.1111/pedi.12323 (2016). 26420304 10.1111/pedi.12323 PMC4703499

[6] Griffiths, W. J. et al. Targeted metabolomics for biomarker discovery. *Angew Chem. Int. Ed. Engl.* **49** (32), 5426–5445. 10.1002/anie.200905579 (2010). 20629054 10.1002/anie.200905579

[7] Aderemi, A. V., Ayeleso, A. O., Oyedapo, O. O. & Mukwevho, E. Metabolomics: A scoping review of its role as a tool for disease biomarker discovery in selected non-communicable diseases. *Metabolites* **11** (7). 10.3390/metabo11070418 (2021). 10.3390/metabo11070418 PMC8305588 34201929

[8] Balderas, C. et al. Plasma and urine metabolic fingerprinting of type 1 diabetic children. *Electrophoresis* **34** (19), 2882–2890. 10.1002/elps.201300062 (2013). 23857511 10.1002/elps.201300062

[9] Dutta, T. et al. Impact of long-term poor and good glycemic control on metabolomics alterations in Type 1 diabetic people. *J. Clin. Endocrinol. Metab.* **101** (3), 1023–1033. 10.1210/jc.2015-2640 (2016). 26796761 10.1210/jc.2015-2640 PMC4803168

[10] Bervoets, L. et al. Metabolic profiling of type 1 diabetes mellitus in children and adolescents: A case-control study. *Diabetol. Metab. Syndr.* **9**, 48. 10.1186/s13098-017-0246-9 (2017). 28674557 10.1186/s13098-017-0246-9 PMC5485735

[11] Sobczak, A. I. S., Pitt, S. J., Smith, T. K., Ajjan, R. A. & Stewart, A. J. Lipidomic profiling of plasma free fatty acids in type-1 diabetes highlights specific changes in lipid metabolism. *Biochim. Biophys. Acta Mol. Cell. Biol. Lipids*. **1866** (1), 158823. 10.1016/j.bbalip.2020.158823 (2021). 33010452 10.1016/j.bbalip.2020.158823 PMC7695620

[12] Marcovecchio, M. L. et al. ACE inhibitors and statins in adolescents with type 1 diabetes. *N Engl. J. Med.* **377** (18), 1733–1745. 10.1056/NEJMoa1703518 (2017). 29091568 10.1056/NEJMoa1703518

[13] Chiesa, S. T. et al. Vascular effects of ACE (angiotensin-converting enzyme) inhibitors and statins in adolescents with type 1 diabetes. *Hypertension* **76** (6), 1734–1743. 10.1161/hypertensionaha.120.15721 (2020). 33100044 10.1161/HYPERTENSIONAHA.120.15721

[14] Kofink, D. et al. Statin effects on metabolic profiles. *Circulation: Cardiovasc. Genet.* **10** (6), e001759. 10.1161/CIRCGENETICS.117.001759 (2017). 10.1161/CIRCGENETICS.117.001759 29237679

[15] O’Keeffe, L. M. et al. Data on trajectories of measures of cardiovascular health in the Avon Longitudinal Study of Parents and Children (ALSPAC). *Data Brief.* **23**, 103687. 10.1016/j.dib.2019.01.035 (2019). 30847378 10.1016/j.dib.2019.01.035 PMC6389726

[16] Eissa, M. A., Mihalopoulos, N. L., Holubkov, R., Dai, S. & Labarthe, D. R. Changes in fasting lipids during puberty. *J. Pediatr.* **170**, 199–205. 10.1016/j.jpeds.2015.11.018 (2016). 26706233 10.1016/j.jpeds.2015.11.018 PMC4769904

[17] Lanktree, M. B., Thériault, S., Walsh, M. & Paré, G. HDL cholesterol, LDL cholesterol, and triglycerides as risk factors for CKD: A mendelian randomization study. *Am. J. Kidney Dis.* **71** (2), 166–172. 10.1053/j.ajkd.2017.06.011 (2018). 28754456 10.1053/j.ajkd.2017.06.011

[18] Huang, S. H. et al. Hyperfiltration affects accuracy of creatinine eGFR measurement. *Clin. J. Am. Soc. Nephrol.* **6** (2), 274–280. 10.2215/cjn.02760310 (2011). 20966120 10.2215/CJN.02760310 PMC3052216

[19] Tan, S., Gunendi, Z., Meray, J. & Yetkin, İ. The evaluation of muscle strength and architecture in type 1 diabetes mellitus: a cross-sectional study. *BMC Endocr. Disord*. **22** (1), 153. 10.1186/s12902-022-01062-y (2022). 35668406 10.1186/s12902-022-01062-y PMC9172182

[20] Pollakova, D. et al. Muscular involvement in long-term type 1 diabetes: Does it represent an underestimated complication? *Nutrition* **112**, 112060. 10.1016/j.nut.2023.112060 (2023). 37267657 10.1016/j.nut.2023.112060

[21] Chuang, G. T., Tsai, I. J. & Tsau, Y. K. Serum creatinine reference limits in pediatric population-A single center electronic health record-based database in Taiwan. *Front. Pediatr.* **9**, 793446. 10.3389/fped.2021.793446 (2021). 35036395 10.3389/fped.2021.793446 PMC8756578

[22] Torres-Tamayo, M. et al. Lipoprotein(a) levels in children and adolescents with diabetes. *Rev. Invest. Clin.* **49** (6), 437–443 (1997). 9580280

[23] Wiltshire, E. J., Hirte, C. & Couper, J. J. Dietary fats do not contribute to hyperlipidemia in children and adolescents with type 1 diabetes. *Diabetes Care*. **26** (5), 1356–1361. 10.2337/diacare.26.5.1356 (2003). 12716788 10.2337/diacare.26.5.1356

[24] Krishnan, S. & Short, K. R. Prevalence and significance of cardiometabolic risk factors in children with type 1 diabetes. *J. Cardiometab Syndr.* **4** (1), 50–56. 10.1111/j.1559-4572.2008.00034.x (2009). 19245517 10.1111/j.1559-4572.2008.00034.x PMC2649740

[25] Chiesa, S. T. et al. Elevated high-density lipoprotein in adolescents with Type 1 diabetes is associated with endothelial dysfunction in the presence of systemic inflammation. *Eur. Heart J.* **40** (43), 3559–3566. 10.1093/eurheartj/ehz114 (2019). 30863865 10.1093/eurheartj/ehz114 PMC6855140

[26] Heier, M. et al. Reduced HDL function in children and young adults with type 1 diabetes. *Cardiovasc. Diabetol.* **16** (1), 85. 10.1186/s12933-017-0570-2 (2017). 28683835 10.1186/s12933-017-0570-2 PMC5501001

[27] Fukao, T., Lopaschuk, G. D. & Mitchell, G. A. Pathways and control of ketone body metabolism: on the fringe of lipid biochemistry. *Prostaglandins Leukot. Essent. Fat. Acids*. **70** (3), 243–251. 10.1016/j.plefa.2003.11.001 (2004). 10.1016/j.plefa.2003.11.001 14769483

[28] Laffel, L. Ketone bodies: A review of physiology, pathophysiology and application of monitoring to diabetes. *Diabetes Metab. Res. Rev.* **15** (6), 412–426. 10.1002/(sici)1520-7560 (1999). 8. 10634967 10.1002/(sici)1520-7560(199911/12)15:6<412::aid-dmrr72>3.0.co;2-8

[29] Hall, S. E., Wastney, M. E., Bolton, T. M., Braaten, J. T. & Berman, M. Ketone body kinetics in humans: The effects of insulin-dependent diabetes, obesity, and starvation. *J. Lipid Res.* **25** (11), 1184–1194 (1984). 6440941

[30] Hegardt, F. G. Mitochondrial 3-hydroxy-3-methylglutaryl-CoA synthase: a control enzyme in ketogenesis. *Biochem. J.* **338** (Pt 3), 569–582 (1999). 10051425 PMC1220089

[31] Close, T. E. et al. Diabetic ketoacidosis elicits systemic inflammation associated with cerebrovascular endothelial cell dysfunction. *Microcirculation* **20** (6), 534–543. 10.1111/micc.12053 (2013). 23441883 10.1111/micc.12053

[32] Kanikarla-Marie, P. & Jain, S. K. Hyperketonemia (acetoacetate) upregulates NADPH oxidase 4 and elevates oxidative stress, ICAM-1, and monocyte adhesivity in endothelial cells. *Cell. Physiol. Biochem.* **35** (1), 364–373. 10.1159/000369702 (2015). 25591777 10.1159/000369702 PMC4309197

[33] Kanikarla-Marie, P. & Jain, S. K. Hyperketonemia and ketosis increase the risk of complications in type 1 diabetes. *Free Radic Biol. Med.* **95**, 268–277. 10.1016/j.freeradbiomed.2016.03.020 (2016). 27036365 10.1016/j.freeradbiomed.2016.03.020 PMC4867238

[34] Hebert, S. L. & Nair, K. S. Protein and energy metabolism in type 1 diabetes. *Clin. Nutr.* **29** (1), 13–17. 10.1016/j.clnu.2009.09.001 (2010). 19788950 10.1016/j.clnu.2009.09.001 PMC2822109

[35] Jitrapakdee, S., Vidal-Puig, A. & Wallace, J. C. Anaplerotic roles of pyruvate carboxylase in mammalian tissues. *Cell. Mol. Life Sci.* **63** (7–8), 843–854. 10.1007/s00018-005-5410-y (2006). 16505973 10.1007/s00018-005-5410-y PMC11136034

[36] Otvos, J. D. et al. GlycA: A composite nuclear magnetic resonance biomarker of systemic inflammation. *Clin. Chem.* **61** (5), 714–723. 10.1373/clinchem.2014.232918 (2015). 25779987 10.1373/clinchem.2014.232918

[37] Connelly, M. A., Otvos, J. D., Shalaurova, I., Playford, M. P. & Mehta, N. N. GlycA, a novel biomarker of systemic inflammation and cardiovascular disease risk. *J. Transl Med.* **15** (1), 219. 10.1186/s12967-017-1321-6 (2017). 29078787 10.1186/s12967-017-1321-6 PMC5658936

[38] Chiesa, S. T. et al. Glycoprotein acetyls: a novel inflammatory biomarker of early cardiovascular risk in the young. *J. Am. Heart Assoc.* **11** (4), e024380. 10.1161/jaha.121.024380 (2022). 35156387 10.1161/JAHA.121.024380 PMC9245818

[39] Adolescent type 1 Diabetes cardio-renal Intervention Trial Research Group. Adolescent type 1 diabetes cardio-renal intervention trial (AdDIT). *BMC Pediatr.* **9**, 79. 10.1186/1471-2431-9-79 (2009). 20017932 10.1186/1471-2431-9-79 PMC2814806

[40] Marcovecchio, M. L. et al. Renal and cardiovascular risk according to tertiles of urinary albumin-to-creatinine ratio: The Adolescent Type 1 Diabetes cardio-renal Intervention Trial (AdDIT). *Diabetes Care*. **41** (9), 1963–1969. 10.2337/dc18-1125 (2018). 30026334 10.2337/dc18-1125

