# Supplementary material for: Effect of statins and ACE inhibitors on the metabolome in the Adolescent Type 1 Diabetes cardio-renal Intervention Trial (AdDIT) cohort
Source: Sci Rep. 2026 Apr 29;16:25681. doi: 10.1038/s41598-026-50044-w (PMC13478414; doi:10.1038/s41598-026-50044-w)
Supplement: Supplementary file 1 [file 41598_2026_50044_MOESM1_ESM.docx]

**Supplementary File**

Effect of Statins and ACE inhibitors on the metabolome in the Adolescent Type 1 Diabetes cardio-renal Intervention Trial (AdDIT)

Prampolini et al 2026

**Table of Contents**

[Abbreviations	3](#_Toc220575100)

[Supplementary Figure 1: Comparison of early adolescent metabolomic profiles in type 1 diabetes vs controls.	9](#_Toc220575101)

[Supplementary Figure 2: Effect of statins on metabolomic profile in T1D	12](#_Toc220575102)

[Supplementary Figure 3: Effect of ACE inhibitors on metabolomic profile in T1D	15](#_Toc220575103)

[Supplementary Table 1: Percent change in metabolite levels over 3.5 years of follow-up of untreated individuals with type 1 diabetes	18](#_Toc220575104)

[Supplementary Table 2: Associations between HbA1c at baseline and during follow up and metabolite levels in late adolescence (17.5 years)	23](#_Toc220575105)

# Abbreviations

| Total-C | Total cholesterol |
| --- | --- |
| non-HDL-C | Total cholesterol minus HDL-C |
| Remnant-C | Remnant cholesterol (non-HDL, non-LDL -cholesterol) |
| VLDL-C | VLDL cholesterol |
| Clinical LDL-C | Clinical LDL cholesterol |
| LDL-C | LDL cholesterol |
| HDL-C | HDL cholesterol |
| Total-TG | Total triglycerides |
| VLDL-TG | Triglycerides in VLDL |
| LDL-TG | Triglycerides in LDL |
| HDL-TG | Triglycerides in HDL |
| Total-PL | Total phospholipids in lipoprotein particles |
| VLDL-PL | Phospholipids in VLDL |
| LDL-PL | Phospholipids in LDL |
| HDL-PL | Phospholipids in HDL |
| Total-CE | Total esterified cholesterol |
| VLDL-CE | Cholesteryl esters in VLDL |
| LDL-CE | Cholesteryl esters in LDL |
| HDL-CE | Cholesteryl esters in HDL |
| Total-FC | Total free cholesterol |
| VLDL-FC | Free cholesterol in VLDL |
| LDL-FC | Free cholesterol in LDL |
| HDL-FC | Free cholesterol in HDL |
| Total-L | Total lipids in lipoprotein particles |
| VLDL-L | Total lipids in VLDL |
| LDL-L | Total lipids in LDL |
| HDL-L | Total lipids in HDL |
| Total-P | Total concentration of lipoprotein particles |
| VLDL-P | Concentration of VLDL particles |
| LDL-P | Concentration of LDL particles |
| HDL-P | Concentration of HDL particles |
| VLDL size | Average diameter for VLDL particles |
| LDL size | Average diameter for LDL particles |
| HDL size | Average diameter for HDL particles |
| Phosphoglyc | Phosphoglycerides |
| TG/PG | Ratio of triglycerides to phosphoglycerides |
| Cholines | Total cholines |
| Phosphatidylc | Phosphatidylcholines |
| Sphingomyelins | Sphingomyelins |
| ApoB | Apolipoprotein B |
| ApoA1 | Apolipoprotein A1 |
| ApoB/ApoA1 | Ratio of apolipoprotein B to apolipoprotein A1 |
| Total-FA | Total fatty acids |
| Unsaturation | Degree of unsaturation |
| Omega-3 | Omega-3 fatty acids |
| Omega-6 | Omega-6 fatty acids |
| PUFA | Polyunsaturated fatty acids |
| MUFA | Monounsaturated fatty acids |
| SFA | Saturated fatty acids |
| LA | Linoleic acid |
| DHA | Docosahexaenoic acid |
| Omega-3 % | Ratio of omega-3 fatty acids to total fatty acids |
| Omega-6 % | Ratio of omega-6 fatty acids to total fatty acids |
| PUFA % | Ratio of polyunsaturated fatty acids to total fatty acids |
| MUFA % | Ratio of monounsaturated fatty acids to total fatty acids |
| SFA % | Ratio of saturated fatty acids to total fatty acids |
| LA % | Ratio of linoleic acid to total fatty acids |
| DHA % | Ratio of docosahexaenoic acid to total fatty acids |
| PUFA/MUFA | Ratio of polyunsaturated fatty acids to monounsaturated fatty acids |
| Omega-6/Omega-3 | Ratio of omega-6 fatty acids to omega-3 fatty acids |
| Ala | Alanine |
| Gln | Glutamine |
| Gly | Glycine |
| His | Histidine |
| Total BCAA | Total concentration of branched-chain amino acids (leucine + isoleucine + valine) |
| Ile | Isoleucine |
| Leu | Leucine |
| Val | Valine |
| Phe | Phenylalanine |
| Tyr | Tyrosine |
| Glucose | Glucose |
| Lactate | Lactate |
| Pyruvate | Pyruvate |
| Citrate | Citrate |
| Glycerol | Glycerol |
| bOHbutyrate | 3-Hydroxybutyrate |
| Acetate | Acetate |
| Acetoacetate | Acetoacetate |
| Acetone | Acetone |
| Creatinine | Creatinine |
| Albumin | Albumin |
| GlycA | Glycoprotein acetyls |
| XXL-VLDL-P | Concentration of chylomicrons and extremely large VLDL particles |
| XXL-VLDL-L | Total lipids in chylomicrons and extremely large VLDL |
| XXL-VLDL-PL | Phospholipids in chylomicrons and extremely large VLDL |
| XXL-VLDL-C | Cholesterol in chylomicrons and extremely large VLDL |
| XXL-VLDL-CE | Cholesteryl esters in chylomicrons and extremely large VLDL |
| XXL-VLDL-FC | Free cholesterol in chylomicrons and extremely large VLDL |
| XXL-VLDL-TG | Triglycerides in chylomicrons and extremely large VLDL |
| XL-VLDL-P | Concentration of very large VLDL particles |
| XL-VLDL-L | Total lipids in very large VLDL |
| XL-VLDL-PL | Phospholipids in very large VLDL |
| XL-VLDL-C | Cholesterol in very large VLDL |
| XL-VLDL-CE | Cholesteryl esters in very large VLDL |
| XL-VLDL-FC | Free cholesterol in very large VLDL |
| XL-VLDL-TG | Triglycerides in very large VLDL |
| L-VLDL-P | Concentration of large VLDL particles |
| L-VLDL-L | Total lipids in large VLDL |
| L-VLDL-PL | Phospholipids in large VLDL |
| L-VLDL-C | Cholesterol in large VLDL |
| L-VLDL-CE | Cholesteryl esters in large VLDL |
| L-VLDL-FC | Free cholesterol in large VLDL |
| L-VLDL-TG | Triglycerides in large VLDL |
| M-VLDL-P | Concentration of medium VLDL particles |
| M-VLDL-L | Total lipids in medium VLDL |
| M-VLDL-PL | Phospholipids in medium VLDL |
| M-VLDL-C | Cholesterol in medium VLDL |
| M-VLDL-CE | Cholesteryl esters in medium VLDL |
| M-VLDL-FC | Free cholesterol in medium VLDL |
| M-VLDL-TG | Triglycerides in medium VLDL |
| S-VLDL-P | Concentration of small VLDL particles |
| S-VLDL-L | Total lipids in small VLDL |
| S-VLDL-PL | Phospholipids in small VLDL |
| S-VLDL-C | Cholesterol in small VLDL |
| S-VLDL-CE | Cholesteryl esters in small VLDL |
| S-VLDL-FC | Free cholesterol in small VLDL |
| S-VLDL-TG | Triglycerides in small VLDL |
| XS-VLDL-P | Concentration of very small VLDL particles |
| XS-VLDL-L | Total lipids in very small VLDL |
| XS-VLDL-PL | Phospholipids in very small VLDL |
| XS-VLDL-C | Cholesterol in very small VLDL |
| XS-VLDL-CE | Cholesteryl esters in very small VLDL |
| XS-VLDL-FC | Free cholesterol in very small VLDL |
| XS-VLDL-TG | Triglycerides in very small VLDL |
| IDL-P | Concentration of IDL particles |
| IDL-L | Total lipids in IDL |
| IDL-PL | Phospholipids in IDL |
| IDL-C | Cholesterol in IDL |
| IDL-CE | Cholesteryl esters in IDL |
| IDL-FC | Free cholesterol in IDL |
| IDL-TG | Triglycerides in IDL |
| L-LDL-P | Concentration of large LDL particles |
| L-LDL-L | Total lipids in large LDL |
| L-LDL-PL | Phospholipids in large LDL |
| L-LDL-C | Cholesterol in large LDL |
| L-LDL-CE | Cholesteryl esters in large LDL |
| L-LDL-FC | Free cholesterol in large LDL |
| L-LDL-TG | Triglycerides in large LDL |
| M-LDL-P | Concentration of medium LDL particles |
| M-LDL-L | Total lipids in medium LDL |
| M-LDL-PL | Phospholipids in medium LDL |
| M-LDL-C | Cholesterol in medium LDL |
| M-LDL-CE | Cholesteryl esters in medium LDL |
| M-LDL-FC | Free cholesterol in medium LDL |
| M-LDL-TG | Triglycerides in medium LDL |
| S-LDL-P | Concentration of small LDL particles |
| S-LDL-L | Total lipids in small LDL |
| S-LDL-PL | Phospholipids in small LDL |
| S-LDL-C | Cholesterol in small LDL |
| S-LDL-CE | Cholesteryl esters in small LDL |
| S-LDL-FC | Free cholesterol in small LDL |
| S-LDL-TG | Triglycerides in small LDL |
| XL-HDL-P | Concentration of very large HDL particles |
| XL-HDL-L | Total lipids in very large HDL |
| XL-HDL-PL | Phospholipids in very large HDL |
| XL-HDL-C | Cholesterol in very large HDL |
| XL-HDL-CE | Cholesteryl esters in very large HDL |
| XL-HDL-FC | Free cholesterol in very large HDL |
| XL-HDL-TG | Triglycerides in very large HDL |
| L-HDL-P | Concentration of large HDL particles |
| L-HDL-L | Total lipids in large HDL |
| L-HDL-PL | Phospholipids in large HDL |
| L-HDL-C | Cholesterol in large HDL |
| L-HDL-CE | Cholesteryl esters in large HDL |
| L-HDL-FC | Free cholesterol in large HDL |
| L-HDL-TG | Triglycerides in large HDL |
| M-HDL-P | Concentration of medium HDL particles |
| M-HDL-L | Total lipids in medium HDL |
| M-HDL-PL | Phospholipids in medium HDL |
| M-HDL-C | Cholesterol in medium HDL |
| M-HDL-CE | Cholesteryl esters in medium HDL |
| M-HDL-FC | Free cholesterol in medium HDL |
| M-HDL-TG | Triglycerides in medium HDL |
| S-HDL-P | Concentration of small HDL particles |
| S-HDL-L | Total lipids in small HDL |
| S-HDL-PL | Phospholipids in small HDL |
| S-HDL-C | Cholesterol in small HDL |
| S-HDL-CE | Cholesteryl esters in small HDL |
| S-HDL-FC | Free cholesterol in small HDL |
| S-HDL-TG | Triglycerides in small HDL |
| XXL-VLDL-PL % | Phospholipids to total lipids ratio in chylomicrons and extremely large VLDL |
| XXL-VLDL-C % | Cholesterol to total lipids ratio in chylomicrons and extremely large VLDL |
| XXL-VLDL-CE % | Cholesteryl esters to total lipids ratio in chylomicrons and extremely large VLDL |
| XXL-VLDL-FC % | Free cholesterol to total lipids ratio in chylomicrons and extremely large VLDL |
| XXL-VLDL-TG % | Triglycerides to total lipids ratio in chylomicrons and extremely large VLDL |
| XL-VLDL-PL % | Phospholipids to total lipids ratio in very large VLDL |
| XL-VLDL-C % | Cholesterol to total lipids ratio in very large VLDL |
| XL-VLDL-CE % | Cholesteryl esters to total lipids ratio in very large VLDL |
| XL-VLDL-FC % | Free cholesterol to total lipids ratio in very large VLDL |
| XL-VLDL-TG % | Triglycerides to total lipids ratio in very large VLDL |
| L-VLDL-PL % | Phospholipids to total lipids ratio in large VLDL |
| L-VLDL-C % | Cholesterol to total lipids ratio in large VLDL |
| L-VLDL-CE % | Cholesteryl esters to total lipids ratio in large VLDL |
| L-VLDL-FC % | Free cholesterol to total lipids ratio in large VLDL |
| L-VLDL-TG % | Triglycerides to total lipids ratio in large VLDL |
| M-VLDL-PL % | Phospholipids to total lipids ratio in medium VLDL |
| M-VLDL-C % | Cholesterol to total lipids ratio in medium VLDL |
| M-VLDL-CE % | Cholesteryl esters to total lipids ratio in medium VLDL |
| M-VLDL-FC % | Free cholesterol to total lipids ratio in medium VLDL |
| M-VLDL-TG % | Triglycerides to total lipids ratio in medium VLDL |
| S-VLDL-PL % | Phospholipids to total lipids ratio in small VLDL |
| S-VLDL-C % | Cholesterol to total lipids ratio in small VLDL |
| S-VLDL-CE % | Cholesteryl esters to total lipids ratio in small VLDL |
| S-VLDL-FC % | Free cholesterol to total lipids ratio in small VLDL |
| S-VLDL-TG % | Triglycerides to total lipids ratio in small VLDL |
| XS-VLDL-PL % | Phospholipids to total lipids ratio in very small VLDL |
| XS-VLDL-C % | Cholesterol to total lipids ratio in very small VLDL |
| XS-VLDL-CE % | Cholesteryl esters to total lipids ratio in very small VLDL |
| XS-VLDL-FC % | Free cholesterol to total lipids ratio in very small VLDL |
| XS-VLDL-TG % | Triglycerides to total lipids ratio in very small VLDL |
| IDL-PL % | Phospholipids to total lipids ratio in IDL |
| IDL-C % | Cholesterol to total lipids ratio in IDL |
| IDL-CE % | Cholesteryl esters to total lipids ratio in IDL |
| IDL-FC % | Free cholesterol to total lipids ratio in IDL |
| IDL-TG % | Triglycerides to total lipids ratio in IDL |
| L-LDL-PL % | Phospholipids to total lipids ratio in large LDL |
| L-LDL-C % | Cholesterol to total lipids ratio in large LDL |
| L-LDL-CE % | Cholesteryl esters to total lipids ratio in large LDL |
| L-LDL-FC % | Free cholesterol to total lipids ratio in large LDL |
| L-LDL-TG % | Triglycerides to total lipids ratio in large LDL |
| M-LDL-PL % | Phospholipids to total lipids ratio in medium LDL |
| M-LDL-C % | Cholesterol to total lipids ratio in medium LDL |
| M-LDL-CE % | Cholesteryl esters to total lipids ratio in medium LDL |
| M-LDL-FC % | Free cholesterol to total lipids ratio in medium LDL |
| M-LDL-TG % | Triglycerides to total lipids ratio in medium LDL |
| S-LDL-PL % | Phospholipids to total lipids ratio in small LDL |
| S-LDL-C % | Cholesterol to total lipids ratio in small LDL |
| S-LDL-CE % | Cholesteryl esters to total lipids ratio in small LDL |
| S-LDL-FC % | Free cholesterol to total lipids ratio in small LDL |
| S-LDL-TG % | Triglycerides to total lipids ratio in small LDL |
| XL-HDL-PL % | Phospholipids to total lipids ratio in very large HDL |
| XL-HDL-C % | Cholesterol to total lipids ratio in very large HDL |
| XL-HDL-CE % | Cholesteryl esters to total lipids ratio in very large HDL |
| XL-HDL-FC % | Free cholesterol to total lipids ratio in very large HDL |
| XL-HDL-TG % | Triglycerides to total lipids ratio in very large HDL |
| L-HDL-PL % | Phospholipids to total lipids ratio in large HDL |
| L-HDL-C % | Cholesterol to total lipids ratio in large HDL |
| L-HDL-CE % | Cholesteryl esters to total lipids ratio in large HDL |
| L-HDL-FC % | Free cholesterol to total lipids ratio in large HDL |
| L-HDL-TG % | Triglycerides to total lipids ratio in large HDL |
| M-HDL-PL % | Phospholipids to total lipids ratio in medium HDL |
| M-HDL-C % | Cholesterol to total lipids ratio in medium HDL |
| M-HDL-CE % | Cholesteryl esters to total lipids ratio in medium HDL |
| M-HDL-FC % | Free cholesterol to total lipids ratio in medium HDL |
| M-HDL-TG % | Triglycerides to total lipids ratio in medium HDL |
| S-HDL-PL % | Phospholipids to total lipids ratio in small HDL |
| S-HDL-C % | Cholesterol to total lipids ratio in small HDL |
| S-HDL-CE % | Cholesteryl esters to total lipids ratio in small HDL |
| S-HDL-FC % | Free cholesterol to total lipids ratio in small HDL |
| S-HDL-TG % | Triglycerides to total lipids ratio in small HDL |

# Supplementary Figure 1: Comparison of early adolescent metabolomic profiles in type 1 diabetes vs controls.

Results show a z- score mean difference (+/- 95%CIs) for each outcome in individuals with type 1 diabetes relative to controls. All models adjusted for age, sex, and BMI.

# Supplementary Figure 2: Effect of statins on metabolomic profile in T1D

Forest plots show mean z-score difference (+/- 95%CIs) at follow-up in individuals taking statins vs those taking placebo. All models adjusted for baseline age, sex, duration of diabetes, HbA1c, initial metabolite level, and country of recruitment.

# Supplementary Figure 3: Effect of ACE inhibitors on metabolomic profile in T1D

Forest plots show mean z-score difference (+/- 95%CIs) at follow-up in individuals taking ACE inhibitors vs those taking placebo. All models adjusted for baseline age, sex, duration of diabetes, HbA1c, initial metabolite level, and country of recruitment.

# Supplementary Table 1: Percent change in metabolite levels over 3.5 years of follow-up of untreated individuals with type 1 diabetes

|  | % Change | Lower 95% CI | Upper 95% CI | p |
| --- | --- | --- | --- | --- |
| non_HDL_C | 13 | 10 | 17 | <0.001 |
| Clinical_LDL_C | 15 | 11 | 18 | <0.001 |
| Total_PL | 5 | 3 | 8 | <0.001 |
| VLDL_PL | 23 | 18 | 28 | <0.001 |
| LDL_PL | 13 | 10 | 16 | <0.001 |
| HDL_PL | -1 | -4 | 2 | 0.444 |
| VLDL_CE | 12 | 7 | 17 | <0.001 |
| LDL_CE | 15 | 12 | 19 | <0.001 |
| HDL_CE | -4 | -6 | -1 | 0.002 |
| VLDL_FC | 21 | 16 | 26 | <0.001 |
| LDL_FC | 12 | 9 | 15 | <0.001 |
| HDL_FC | -1 | -4 | 2 | 0.478 |
| Total_L | 9 | 6 | 12 | <0.001 |
| VLDL_L | 26 | 20 | 31 | <0.001 |
| LDL_L | 14 | 11 | 17 | <0.001 |
| HDL_L | -2 | -4 | 1 | 0.229 |
| Total_P | 2 | 0 | 4 | 0.056 |
| VLDL_P | 17 | 13 | 21 | <0.001 |
| LDL_P | 11 | 8 | 14 | <0.001 |
| HDL_P | 1 | -1 | 4 | 0.234 |
| VLDL_size | 2 | 1 | 2 | <0.001 |
| LDL_size | 0 | 0 | 0 | <0.001 |
| HDL_size | -1 | -1 | -1 | <0.001 |
| TG_by_PG | 21 | 17 | 25 | <0.001 |
| Unsaturation | -2 | -2 | -1 | <0.001 |
| Omega_3_pct | -2 | -5 | 2 | 0.349 |
| Omega_6_pct | -3 | -4 | -2 | <0.001 |
| PUFA_pct | -3 | -4 | -2 | <0.001 |
| MUFA_pct | 4 | 2 | 5 | <0.001 |
| SFA_pct | 1 | 1 | 2 | <0.001 |
| LA_pct | -1 | -2 | 0 | 0.041 |
| DHA_pct | -5 | -8 | -2 | 0.001 |
| PUFA_by_MUFA | -6 | -8 | -4 | <0.001 |
| Omega_6_by_Omega_3 | -2 | -5 | 2 | 0.341 |
| Gly | -15 | -19 | -11 | <0.001 |
| Total_BCAA | -6 | -9 | -3 | <0.001 |
| Acetone | -15 | -24 | -6 | <0.001 |
| XXL_VLDL_P | 57 | 31 | 83 | 0.001 |
| XXL_VLDL_L | 12 | -9 | 32 | 0.292 |
| XXL_VLDL_PL | 77 | 39 | 115 | 0.003 |
| XXL_VLDL_C | 2 | -14 | 18 | 0.801 |
| XXL_VLDL_CE | -4 | -19 | 12 | 0.642 |
| XXL_VLDL_FC | 15 | -4 | 34 | 0.143 |
| XXL_VLDL_TG | -24 | -77 | 29 | 0.316 |
| XL_VLDL_P | 82 | 69 | 94 | <0.001 |
| XL_VLDL_L | 65 | 54 | 77 | <0.001 |
| XL_VLDL_PL | 122 | 97 | 146 | <0.001 |
| XL_VLDL_C | 33 | 23 | 43 | <0.001 |
| XL_VLDL_CE | 24 | 16 | 33 | <0.001 |
| XL_VLDL_FC | 54 | 41 | 67 | <0.001 |
| XL_VLDL_TG | 112 | 92 | 131 | <0.001 |
| L_VLDL_P | 51 | 43 | 59 | <0.001 |
| L_VLDL_L | 52 | 44 | 59 | <0.001 |
| L_VLDL_PL | 110 | 91 | 129 | <0.001 |
| L_VLDL_C | 43 | 34 | 51 | <0.001 |
| L_VLDL_CE | 36 | 27 | 44 | <0.001 |
| L_VLDL_FC | 51 | 42 | 60 | <0.001 |
| L_VLDL_TG | 53 | 45 | 60 | <0.001 |
| M_VLDL_P | 22 | 17 | 26 | <0.001 |
| M_VLDL_L | 26 | 21 | 30 | <0.001 |
| M_VLDL_PL | 19 | 9 | 29 | 0.001 |
| M_VLDL_C | 15 | 10 | 20 | <0.001 |
| M_VLDL_CE | 11 | 6 | 16 | <0.001 |
| M_VLDL_FC | 21 | 16 | 26 | <0.001 |
| M_VLDL_TG | 34 | 29 | 39 | <0.001 |
| S_VLDL_P | 18 | 13 | 22 | <0.001 |
| S_VLDL_L | 17 | 13 | 22 | <0.001 |
| S_VLDL_PL | 16 | 12 | 20 | <0.001 |
| S_VLDL_C | 13 | 8 | 17 | <0.001 |
| S_VLDL_CE | 11 | 6 | 16 | <0.001 |
| S_VLDL_FC | 15 | 11 | 19 | <0.001 |
| S_VLDL_TG | 23 | 19 | 28 | <0.001 |
| XS_VLDL_P | 9 | 6 | 12 | <0.001 |
| XS_VLDL_L | 8 | 5 | 12 | <0.001 |
| XS_VLDL_PL | 8 | 4 | 11 | <0.001 |
| XS_VLDL_C | 7 | 4 | 11 | <0.001 |
| XS_VLDL_CE | 7 | 4 | 10 | <0.001 |
| XS_VLDL_FC | 8 | 5 | 12 | <0.001 |
| XS_VLDL_TG | 12 | 8 | 16 | <0.001 |
| IDL_P | 8 | 5 | 10 | <0.001 |
| IDL_L | 9 | 6 | 12 | <0.001 |
| IDL_PL | 7 | 4 | 10 | <0.001 |
| IDL_C | 9 | 6 | 12 | <0.001 |
| IDL_CE | 10 | 7 | 13 | <0.001 |
| IDL_FC | 6 | 3 | 9 | <0.001 |
| IDL_TG | 9 | 5 | 13 | <0.001 |
| L_LDL_P | 9 | 6 | 12 | <0.001 |
| L_LDL_L | 13 | 9 | 16 | <0.001 |
| L_LDL_PL | 12 | 9 | 14 | <0.001 |
| L_LDL_C | 13 | 10 | 16 | <0.001 |
| L_LDL_CE | 14 | 10 | 17 | <0.001 |
| L_LDL_FC | 11 | 8 | 14 | <0.001 |
| L_LDL_TG | 13 | 9 | 16 | <0.001 |
| M_LDL_P | 16 | 13 | 19 | <0.001 |
| M_LDL_L | 18 | 15 | 22 | <0.001 |
| M_LDL_PL | 17 | 14 | 21 | <0.001 |
| M_LDL_C | 19 | 15 | 22 | <0.001 |
| M_LDL_CE | 21 | 17 | 24 | <0.001 |
| M_LDL_FC | 15 | 12 | 18 | <0.001 |
| M_LDL_TG | 17 | 13 | 21 | <0.001 |
| S_LDL_P | 12 | 10 | 15 | <0.001 |
| S_LDL_L | 14 | 11 | 17 | <0.001 |
| S_LDL_PL | 13 | 11 | 15 | <0.001 |
| S_LDL_C | 14 | 11 | 17 | <0.001 |
| S_LDL_CE | 14 | 11 | 17 | <0.001 |
| S_LDL_FC | 13 | 10 | 16 | <0.001 |
| S_LDL_TG | 17 | 13 | 22 | <0.001 |
| XL_HDL_P | -6 | -10 | -3 | <0.001 |
| XL_HDL_L | -10 | -14 | -6 | <0.001 |
| XL_HDL_PL | -13 | -22 | -4 | 0.003 |
| XL_HDL_C | -8 | -12 | -5 | <0.001 |
| XL_HDL_CE | -10 | -13 | -6 | <0.001 |
| XL_HDL_FC | -4 | -7 | -1 | 0.008 |
| XL_HDL_TG | 10 | 5 | 15 | <0.001 |
| L_HDL_P | -12 | -17 | -8 | <0.001 |
| L_HDL_L | -11 | -15 | -7 | <0.001 |
| L_HDL_PL | -10 | -14 | -6 | <0.001 |
| L_HDL_C | -14 | -18 | -9 | <0.001 |
| L_HDL_CE | -14 | -19 | -10 | <0.001 |
| L_HDL_FC | -11 | -16 | -7 | <0.001 |
| L_HDL_TG | 7 | 0 | 13 | 0.049 |
| M_HDL_P | -1 | -4 | 2 | 0.339 |
| M_HDL_L | -1 | -3 | 2 | 0.68 |
| M_HDL_PL | 0 | -3 | 3 | 0.955 |
| M_HDL_C | -2 | -5 | 1 | 0.15 |
| M_HDL_CE | -3 | -5 | 0 | 0.08 |
| M_HDL_FC | 0 | -4 | 3 | 0.841 |
| M_HDL_TG | 15 | 9 | 20 | <0.001 |
| S_HDL_P | 5 | 3 | 7 | <0.001 |
| S_HDL_L | 5 | 3 | 7 | <0.001 |
| S_HDL_PL | 5 | 3 | 7 | <0.001 |
| S_HDL_C | 5 | 2 | 7 | <0.001 |
| S_HDL_CE | 4 | 2 | 6 | <0.001 |
| S_HDL_FC | 6 | 4 | 8 | <0.001 |
| S_HDL_TG | 19 | 15 | 24 | <0.001 |
| XXL_VLDL_PL_pct | 75 | 53 | 98 | <0.001 |
| XXL_VLDL_C_pct | -12 | -20 | -5 | <0.001 |
| XXL_VLDL_CE_pct | -17 | -26 | -9 | <0.001 |
| XXL_VLDL_FC_pct | 0 | -7 | 7 | 0.994 |
| XXL_VLDL_TG_pct | -9 | -37 | 18 | 0.495 |
| XL_VLDL_PL_pct | 26 | 14 | 38 | <0.001 |
| XL_VLDL_C_pct | -20 | -24 | -16 | <0.001 |
| XL_VLDL_CE_pct | -26 | -31 | -21 | <0.001 |
| XL_VLDL_FC_pct | -7 | -11 | -4 | <0.001 |
| XL_VLDL_TG_pct | 30 | 17 | 43 | <0.001 |
| L_VLDL_PL_pct | 38 | 24 | 53 | <0.001 |
| L_VLDL_C_pct | -6 | -9 | -3 | <0.001 |
| L_VLDL_CE_pct | -10 | -14 | -7 | <0.001 |
| L_VLDL_FC_pct | 0 | -3 | 3 | 0.915 |
| L_VLDL_TG_pct | 1 | -2 | 3 | 0.591 |
| M_VLDL_PL_pct | -5 | -12 | 2 | 0.138 |
| M_VLDL_C_pct | -8 | -10 | -6 | <0.001 |
| M_VLDL_CE_pct | -12 | -15 | -8 | <0.001 |
| M_VLDL_FC_pct | -4 | -5 | -2 | <0.001 |
| M_VLDL_TG_pct | 7 | 5 | 8 | <0.001 |
| S_VLDL_PL_pct | -1 | -2 | 0 | 0.012 |
| S_VLDL_C_pct | -4 | -5 | -3 | <0.001 |
| S_VLDL_CE_pct | -5 | -7 | -4 | <0.001 |
| S_VLDL_FC_pct | -2 | -4 | 0 | 0.012 |
| S_VLDL_TG_pct | 5 | 3 | 7 | <0.001 |
| XS_VLDL_PL_pct | -1 | -1 | 0 | 0.051 |
| XS_VLDL_C_pct | -1 | -2 | 0 | 0.077 |
| XS_VLDL_CE_pct | -1 | -3 | 0 | 0.052 |
| XS_VLDL_FC_pct | 0 | 0 | 0 | 0.958 |
| XS_VLDL_TG_pct | 3 | 1 | 5 | 0.003 |
| IDL_PL_pct | -2 | -2 | -1 | <0.001 |
| IDL_C_pct | 0 | 0 | 1 | 0.055 |
| IDL_CE_pct | 1 | 1 | 2 | <0.001 |
| IDL_FC_pct | -2 | -3 | -1 | <0.001 |
| IDL_TG_pct | 0 | -3 | 3 | 0.874 |
| L_LDL_PL_pct | -1 | -1 | -1 | <0.001 |
| L_LDL_C_pct | 0 | 0 | 0 | 0.064 |
| L_LDL_CE_pct | 1 | 1 | 1 | <0.001 |
| L_LDL_FC_pct | -2 | -2 | -1 | <0.001 |
| L_LDL_TG_pct | 0 | -3 | 3 | 0.994 |
| M_LDL_PL_pct | -1 | -1 | 0 | <0.001 |
| M_LDL_C_pct | 0 | 0 | 1 | 0.004 |
| M_LDL_CE_pct | 2 | 1 | 2 | <0.001 |
| M_LDL_FC_pct | -3 | -4 | -2 | <0.001 |
| M_LDL_TG_pct | -2 | -4 | 1 | 0.241 |
| S_LDL_PL_pct | -1 | -1 | 0 | 0.089 |
| S_LDL_C_pct | 0 | 0 | 0 | 0.677 |
| S_LDL_CE_pct | 0 | 0 | 1 | 0.252 |
| S_LDL_FC_pct | -1 | -2 | 0 | 0.236 |
| S_LDL_TG_pct | 3 | 0 | 6 | 0.053 |
| XL_HDL_PL_pct | -3 | -9 | 3 | 0.302 |
| XL_HDL_C_pct | 2 | 1 | 3 | 0.001 |
| XL_HDL_CE_pct | 1 | -1 | 2 | 0.431 |
| XL_HDL_FC_pct | 6 | 4 | 8 | <0.001 |
| XL_HDL_TG_pct | 23 | 17 | 28 | <0.001 |
| L_HDL_PL_pct | 2 | 1 | 2 | <0.001 |
| L_HDL_C_pct | -3 | -4 | -2 | <0.001 |
| L_HDL_CE_pct | -4 | -4 | -3 | <0.001 |
| L_HDL_FC_pct | 0 | -1 | 1 | 0.548 |
| L_HDL_TG_pct | 20 | 15 | 25 | <0.001 |
| M_HDL_PL_pct | 1 | 0 | 1 | <0.001 |
| M_HDL_C_pct | -2 | -2 | -1 | <0.001 |
| M_HDL_CE_pct | -2 | -2 | -1 | <0.001 |
| M_HDL_FC_pct | 0 | -1 | 1 | 0.564 |
| M_HDL_TG_pct | 15 | 10 | 20 | <0.001 |
| S_HDL_PL_pct | 0 | 0 | 0 | 0.019 |
| S_HDL_C_pct | -1 | -1 | 0 | 0.024 |
| S_HDL_CE_pct | -1 | -2 | 0 | 0.005 |
| S_HDL_FC_pct | 1 | 0 | 1 | 0.011 |
| S_HDL_TG_pct | 13 | 10 | 17 | <0.001 |

All models adjusted for baseline age, sex, BMI, initial metabolite level, and time between visits.

# Supplementary Table 2: Associations between HbA1c at baseline and during follow up and metabolite levels in late adolescence (17.5 years)

|  | Baseline | | | | Increase over time | | | |
| --- | --- | --- | --- | --- | --- | --- | --- | --- |
|  | Beta | Lower 95% CI | Upper 95% CI | p | Beta | Lower 95% CI | Upper 95% CI | p |
| non_HDL_C | 0.04 | -0.03 | 0.11 | 0.227 | 0.10 | 0.06 | 0.15 | <0.001 |
| Clinical_LDL_C | 0.05 | -0.01 | 0.11 | 0.126 | 0.09 | 0.04 | 0.13 | 0.001 |
| Total_PL | 0.05 | -0.02 | 0.12 | 0.166 | 0.11 | 0.06 | 0.16 | <0.001 |
| VLDL_PL | 0.01 | -0.06 | 0.09 | 0.777 | 0.15 | 0.10 | 0.21 | <0.001 |
| LDL_PL | 0.06 | -0.01 | 0.13 | 0.073 | 0.10 | 0.05 | 0.15 | <0.001 |
| HDL_PL | 0.03 | -0.03 | 0.09 | 0.362 | 0.05 | 0.00 | 0.10 | 0.037 |
| VLDL_CE | 0.01 | -0.05 | 0.08 | 0.745 | 0.12 | 0.07 | 0.17 | <0.001 |
| LDL_CE | 0.06 | -0.01 | 0.12 | 0.098 | 0.10 | 0.05 | 0.15 | <0.001 |
| HDL_CE | 0.03 | -0.03 | 0.09 | 0.345 | 0.02 | -0.02 | 0.07 | 0.325 |
| VLDL_FC | 0.01 | -0.06 | 0.08 | 0.758 | 0.14 | 0.09 | 0.20 | <0.001 |
| LDL_FC | 0.05 | -0.01 | 0.11 | 0.129 | 0.07 | 0.03 | 0.12 | 0.003 |
| HDL_FC | 0.02 | -0.04 | 0.08 | 0.505 | 0.04 | 0.00 | 0.09 | 0.055 |
| Total_L | 0.05 | -0.02 | 0.12 | 0.174 | 0.13 | 0.07 | 0.18 | <0.001 |
| VLDL_L | 0.01 | -0.07 | 0.09 | 0.828 | 0.15 | 0.10 | 0.21 | <0.001 |
| LDL_L | 0.06 | -0.01 | 0.12 | 0.087 | 0.10 | 0.05 | 0.15 | <0.001 |
| HDL_L | 0.03 | -0.03 | 0.09 | 0.363 | 0.05 | 0.00 | 0.10 | 0.05 |
| Total_P | 0.07 | 0.00 | 0.14 | 0.037 | 0.08 | 0.03 | 0.14 | 0.002 |
| VLDL_P | 0.02 | -0.06 | 0.09 | 0.654 | 0.14 | 0.09 | 0.20 | <0.001 |
| LDL_P | 0.03 | -0.03 | 0.10 | 0.301 | 0.11 | 0.06 | 0.16 | <0.001 |
| HDL_P | 0.07 | 0.00 | 0.14 | 0.044 | 0.07 | 0.02 | 0.13 | 0.006 |
| VLDL_size | -0.01 | -0.07 | 0.06 | 0.829 | 0.07 | 0.02 | 0.12 | 0.004 |
| LDL_size | 0.00 | -0.07 | 0.08 | 0.926 | -0.06 | -0.12 | 0.00 | 0.053 |
| HDL_size | -0.01 | -0.06 | 0.04 | 0.736 | -0.02 | -0.06 | 0.02 | 0.283 |
| TG_by_PG | -0.03 | -0.10 | 0.05 | 0.47 | 0.10 | 0.04 | 0.15 | <0.001 |
| Unsaturation | 0.00 | -0.06 | 0.06 | 0.996 | -0.06 | -0.10 | -0.01 | 0.016 |
| Omega_3_pct | -0.01 | -0.07 | 0.04 | 0.649 | 0.01 | -0.04 | 0.05 | 0.759 |
| Omega_6_pct | -0.01 | -0.08 | 0.07 | 0.817 | -0.12 | -0.17 | -0.06 | <0.001 |
| PUFA_pct | -0.02 | -0.09 | 0.06 | 0.648 | -0.11 | -0.17 | -0.06 | <0.001 |
| MUFA_pct | 0.00 | -0.06 | 0.07 | 0.958 | 0.12 | 0.07 | 0.17 | <0.001 |
| SFA_pct | 0.02 | -0.05 | 0.09 | 0.62 | 0.04 | -0.01 | 0.09 | 0.158 |
| LA_pct | -0.01 | -0.08 | 0.05 | 0.706 | -0.05 | -0.10 | 0.00 | 0.045 |
| DHA_pct | 0.03 | -0.04 | 0.09 | 0.433 | -0.04 | -0.09 | 0.01 | 0.097 |
| PUFA_by_MUFA | -0.01 | -0.07 | 0.06 | 0.841 | -0.11 | -0.16 | -0.06 | <0.001 |
| Omega_6_by_Omega_3 | 0.02 | -0.04 | 0.07 | 0.575 | -0.03 | -0.07 | 0.02 | 0.249 |
| Gly | -0.02 | -0.09 | 0.05 | 0.573 | 0.01 | -0.04 | 0.07 | 0.697 |
| Total_BCAA | 0.05 | -0.02 | 0.12 | 0.183 | 0.05 | -0.01 | 0.10 | 0.104 |
| Acetone | 0.17 | 0.07 | 0.27 | 0.001 | 0.07 | -0.01 | 0.15 | 0.104 |
| XXL_VLDL_P | -0.01 | -0.09 | 0.07 | 0.828 | 0.16 | 0.10 | 0.22 | <0.001 |
| XXL_VLDL_L | -0.01 | -0.09 | 0.07 | 0.799 | 0.16 | 0.09 | 0.22 | <0.001 |
| XXL_VLDL_PL | -0.01 | -0.09 | 0.07 | 0.856 | 0.16 | 0.10 | 0.22 | <0.001 |
| XXL_VLDL_C | 0.00 | -0.08 | 0.08 | 0.987 | 0.16 | 0.10 | 0.22 | <0.001 |
| XXL_VLDL_CE | 0.01 | -0.07 | 0.08 | 0.858 | 0.16 | 0.10 | 0.21 | <0.001 |
| XXL_VLDL_FC | -0.01 | -0.09 | 0.07 | 0.884 | 0.16 | 0.10 | 0.22 | <0.001 |
| XXL_VLDL_TG | -0.01 | -0.10 | 0.07 | 0.776 | 0.15 | 0.09 | 0.21 | <0.001 |
| XL_VLDL_P | 0.00 | -0.08 | 0.08 | 0.957 | 0.15 | 0.09 | 0.21 | <0.001 |
| XL_VLDL_L | 0.00 | -0.08 | 0.08 | 0.974 | 0.15 | 0.09 | 0.21 | <0.001 |
| XL_VLDL_PL | 0.00 | -0.08 | 0.08 | 0.929 | 0.15 | 0.09 | 0.21 | <0.001 |
| XL_VLDL_C | 0.00 | -0.07 | 0.08 | 0.912 | 0.14 | 0.09 | 0.20 | <0.001 |
| XL_VLDL_CE | 0.01 | -0.06 | 0.08 | 0.757 | 0.13 | 0.08 | 0.19 | <0.001 |
| XL_VLDL_FC | 0.00 | -0.08 | 0.08 | 0.95 | 0.15 | 0.09 | 0.20 | <0.001 |
| XL_VLDL_TG | 0.00 | -0.08 | 0.08 | 0.967 | 0.14 | 0.08 | 0.20 | <0.001 |
| L_VLDL_P | 0.01 | -0.07 | 0.09 | 0.875 | 0.14 | 0.09 | 0.20 | <0.001 |
| L_VLDL_L | 0.00 | -0.07 | 0.08 | 0.916 | 0.14 | 0.08 | 0.20 | <0.001 |
| L_VLDL_PL | 0.00 | -0.07 | 0.08 | 0.923 | 0.14 | 0.09 | 0.20 | <0.001 |
| L_VLDL_C | 0.00 | -0.07 | 0.08 | 0.917 | 0.14 | 0.08 | 0.19 | <0.001 |
| L_VLDL_CE | 0.00 | -0.07 | 0.07 | 0.949 | 0.13 | 0.07 | 0.18 | <0.001 |
| L_VLDL_FC | 0.01 | -0.07 | 0.08 | 0.892 | 0.14 | 0.08 | 0.20 | <0.001 |
| L_VLDL_TG | 0.01 | -0.07 | 0.09 | 0.887 | 0.13 | 0.07 | 0.19 | <0.001 |
| M_VLDL_P | 0.02 | -0.05 | 0.09 | 0.576 | 0.13 | 0.07 | 0.18 | <0.001 |
| M_VLDL_L | 0.02 | -0.05 | 0.09 | 0.588 | 0.13 | 0.08 | 0.19 | <0.001 |
| M_VLDL_PL | 0.02 | -0.05 | 0.09 | 0.606 | 0.13 | 0.08 | 0.18 | <0.001 |
| M_VLDL_C | 0.02 | -0.04 | 0.08 | 0.583 | 0.10 | 0.05 | 0.14 | <0.001 |
| M_VLDL_CE | 0.02 | -0.04 | 0.07 | 0.58 | 0.08 | 0.03 | 0.12 | 0.001 |
| M_VLDL_FC | 0.02 | -0.05 | 0.08 | 0.6 | 0.12 | 0.07 | 0.17 | <0.001 |
| M_VLDL_TG | 0.02 | -0.06 | 0.10 | 0.641 | 0.14 | 0.08 | 0.20 | <0.001 |
| S_VLDL_P | 0.03 | -0.05 | 0.11 | 0.44 | 0.16 | 0.10 | 0.21 | <0.001 |
| S_VLDL_L | 0.03 | -0.04 | 0.11 | 0.381 | 0.15 | 0.10 | 0.21 | <0.001 |
| S_VLDL_PL | 0.03 | -0.04 | 0.10 | 0.342 | 0.14 | 0.09 | 0.19 | <0.001 |
| S_VLDL_C | 0.03 | -0.04 | 0.09 | 0.396 | 0.13 | 0.08 | 0.18 | <0.001 |
| S_VLDL_CE | 0.03 | -0.04 | 0.09 | 0.421 | 0.13 | 0.08 | 0.18 | <0.001 |
| S_VLDL_FC | 0.03 | -0.03 | 0.10 | 0.354 | 0.12 | 0.07 | 0.17 | <0.001 |
| S_VLDL_TG | 0.03 | -0.05 | 0.11 | 0.473 | 0.16 | 0.10 | 0.22 | <0.001 |
| XS_VLDL_P | 0.00 | -0.06 | 0.07 | 0.906 | 0.12 | 0.07 | 0.17 | <0.001 |
| XS_VLDL_L | 0.00 | -0.06 | 0.07 | 0.926 | 0.11 | 0.07 | 0.16 | <0.001 |
| XS_VLDL_PL | 0.00 | -0.06 | 0.07 | 0.923 | 0.11 | 0.06 | 0.16 | <0.001 |
| XS_VLDL_C | -0.01 | -0.07 | 0.06 | 0.839 | 0.08 | 0.04 | 0.13 | <0.001 |
| XS_VLDL_CE | -0.01 | -0.07 | 0.05 | 0.779 | 0.07 | 0.03 | 0.12 | 0.001 |
| XS_VLDL_FC | 0.00 | -0.07 | 0.07 | 0.994 | 0.11 | 0.06 | 0.15 | <0.001 |
| XS_VLDL_TG | 0.03 | -0.05 | 0.11 | 0.455 | 0.16 | 0.10 | 0.22 | <0.001 |
| IDL_P | 0.02 | -0.04 | 0.08 | 0.556 | 0.09 | 0.04 | 0.13 | <0.001 |
| IDL_L | 0.03 | -0.03 | 0.09 | 0.373 | 0.08 | 0.04 | 0.13 | <0.001 |
| IDL_PL | 0.02 | -0.04 | 0.08 | 0.503 | 0.09 | 0.04 | 0.13 | <0.001 |
| IDL_C | 0.03 | -0.03 | 0.09 | 0.362 | 0.07 | 0.02 | 0.12 | 0.003 |
| IDL_CE | 0.03 | -0.03 | 0.09 | 0.337 | 0.07 | 0.02 | 0.12 | 0.003 |
| IDL_FC | 0.02 | -0.04 | 0.08 | 0.446 | 0.06 | 0.02 | 0.11 | 0.007 |
| IDL_TG | 0.03 | -0.05 | 0.11 | 0.434 | 0.16 | 0.10 | 0.21 | <0.001 |
| L_LDL_P | 0.03 | -0.03 | 0.09 | 0.315 | 0.10 | 0.06 | 0.15 | <0.001 |
| L_LDL_L | 0.06 | -0.01 | 0.12 | 0.094 | 0.10 | 0.05 | 0.15 | <0.001 |
| L_LDL_PL | 0.06 | -0.01 | 0.12 | 0.089 | 0.09 | 0.04 | 0.14 | <0.001 |
| L_LDL_C | 0.05 | -0.01 | 0.12 | 0.107 | 0.09 | 0.04 | 0.14 | 0.001 |
| L_LDL_CE | 0.06 | -0.01 | 0.12 | 0.092 | 0.09 | 0.04 | 0.14 | <0.001 |
| L_LDL_FC | 0.04 | -0.02 | 0.11 | 0.178 | 0.07 | 0.02 | 0.12 | 0.004 |
| L_LDL_TG | 0.05 | -0.03 | 0.13 | 0.185 | 0.16 | 0.11 | 0.22 | <0.001 |
| M_LDL_P | 0.03 | -0.03 | 0.10 | 0.33 | 0.12 | 0.06 | 0.17 | <0.001 |
| M_LDL_L | 0.06 | -0.01 | 0.13 | 0.077 | 0.11 | 0.06 | 0.17 | <0.001 |
| M_LDL_PL | 0.07 | 0.00 | 0.14 | 0.042 | 0.11 | 0.06 | 0.16 | <0.001 |
| M_LDL_C | 0.06 | -0.01 | 0.12 | 0.105 | 0.11 | 0.06 | 0.16 | <0.001 |
| M_LDL_CE | 0.05 | -0.02 | 0.12 | 0.134 | 0.11 | 0.06 | 0.17 | <0.001 |
| M_LDL_FC | 0.06 | 0.00 | 0.13 | 0.067 | 0.08 | 0.03 | 0.13 | 0.001 |
| M_LDL_TG | 0.06 | -0.02 | 0.14 | 0.14 | 0.18 | 0.12 | 0.24 | <0.001 |
| S_LDL_P | 0.04 | -0.02 | 0.11 | 0.202 | 0.13 | 0.07 | 0.18 | <0.001 |
| S_LDL_L | 0.05 | -0.01 | 0.12 | 0.126 | 0.12 | 0.07 | 0.17 | <0.001 |
| S_LDL_PL | 0.05 | -0.02 | 0.11 | 0.15 | 0.11 | 0.06 | 0.16 | <0.001 |
| S_LDL_C | 0.05 | -0.01 | 0.12 | 0.118 | 0.11 | 0.06 | 0.16 | <0.001 |
| S_LDL_CE | 0.05 | -0.02 | 0.12 | 0.129 | 0.12 | 0.07 | 0.17 | <0.001 |
| S_LDL_FC | 0.05 | -0.01 | 0.11 | 0.117 | 0.07 | 0.02 | 0.12 | 0.006 |
| S_LDL_TG | 0.03 | -0.05 | 0.11 | 0.474 | 0.18 | 0.12 | 0.25 | <0.001 |
| XL_HDL_P | -0.02 | -0.07 | 0.03 | 0.385 | 0.00 | -0.04 | 0.04 | 0.891 |
| XL_HDL_L | -0.02 | -0.07 | 0.03 | 0.342 | -0.02 | -0.06 | 0.02 | 0.397 |
| XL_HDL_PL | -0.03 | -0.08 | 0.02 | 0.3 | -0.02 | -0.06 | 0.02 | 0.234 |
| XL_HDL_C | -0.02 | -0.07 | 0.03 | 0.412 | -0.02 | -0.06 | 0.02 | 0.372 |
| XL_HDL_CE | -0.02 | -0.07 | 0.03 | 0.533 | -0.01 | -0.05 | 0.02 | 0.482 |
| XL_HDL_FC | -0.04 | -0.10 | 0.01 | 0.119 | -0.03 | -0.08 | 0.01 | 0.128 |
| XL_HDL_TG | -0.01 | -0.08 | 0.06 | 0.7 | 0.13 | 0.07 | 0.18 | <0.001 |
| L_HDL_P | -0.01 | -0.06 | 0.04 | 0.805 | 0.00 | -0.04 | 0.04 | 0.85 |
| L_HDL_L | 0.00 | -0.06 | 0.05 | 0.85 | 0.00 | -0.04 | 0.04 | 0.984 |
| L_HDL_PL | 0.00 | -0.05 | 0.05 | 0.921 | 0.00 | -0.04 | 0.04 | 0.864 |
| L_HDL_C | -0.01 | -0.06 | 0.05 | 0.836 | -0.01 | -0.05 | 0.03 | 0.681 |
| L_HDL_CE | 0.00 | -0.06 | 0.05 | 0.859 | -0.01 | -0.05 | 0.03 | 0.607 |
| L_HDL_FC | -0.01 | -0.06 | 0.04 | 0.755 | 0.00 | -0.04 | 0.04 | 0.962 |
| L_HDL_TG | -0.02 | -0.09 | 0.04 | 0.508 | 0.08 | 0.03 | 0.13 | 0.003 |
| M_HDL_P | 0.05 | -0.02 | 0.11 | 0.164 | 0.06 | 0.01 | 0.11 | 0.026 |
| M_HDL_L | 0.05 | -0.02 | 0.12 | 0.139 | 0.06 | 0.01 | 0.11 | 0.015 |
| M_HDL_PL | 0.05 | -0.02 | 0.12 | 0.141 | 0.07 | 0.02 | 0.12 | 0.009 |
| M_HDL_C | 0.05 | -0.02 | 0.11 | 0.136 | 0.04 | -0.01 | 0.09 | 0.084 |
| M_HDL_CE | 0.05 | -0.01 | 0.11 | 0.125 | 0.04 | -0.01 | 0.09 | 0.113 |
| M_HDL_FC | 0.04 | -0.02 | 0.11 | 0.2 | 0.06 | 0.01 | 0.11 | 0.025 |
| M_HDL_TG | 0.01 | -0.06 | 0.09 | 0.757 | 0.12 | 0.06 | 0.17 | <0.001 |
| S_HDL_P | 0.11 | 0.04 | 0.18 | 0.003 | 0.10 | 0.04 | 0.15 | 0.001 |
| S_HDL_L | 0.10 | 0.03 | 0.17 | 0.006 | 0.11 | 0.05 | 0.17 | <0.001 |
| S_HDL_PL | 0.09 | 0.02 | 0.17 | 0.012 | 0.11 | 0.05 | 0.16 | <0.001 |
| S_HDL_C | 0.11 | 0.04 | 0.18 | 0.002 | 0.09 | 0.03 | 0.14 | 0.002 |
| S_HDL_CE | 0.11 | 0.04 | 0.18 | 0.002 | 0.08 | 0.02 | 0.13 | 0.007 |
| S_HDL_FC | 0.10 | 0.02 | 0.17 | 0.01 | 0.11 | 0.06 | 0.17 | <0.001 |
| S_HDL_TG | 0.03 | -0.05 | 0.11 | 0.452 | 0.15 | 0.09 | 0.20 | <0.001 |
| XXL_VLDL_PL_pct | 0.01 | -0.06 | 0.08 | 0.738 | 0.05 | 0.00 | 0.10 | 0.073 |
| XXL_VLDL_C_pct | 0.04 | -0.03 | 0.12 | 0.261 | -0.01 | -0.07 | 0.05 | 0.792 |
| XXL_VLDL_CE_pct | 0.04 | -0.03 | 0.12 | 0.246 | -0.01 | -0.07 | 0.05 | 0.693 |
| XXL_VLDL_FC_pct | 0.02 | -0.05 | 0.10 | 0.54 | 0.01 | -0.05 | 0.07 | 0.791 |
| XXL_VLDL_TG_pct | -0.05 | -0.12 | 0.03 | 0.214 | -0.01 | -0.06 | 0.05 | 0.79 |
| XL_VLDL_PL_pct | 0.01 | -0.05 | 0.07 | 0.828 | 0.00 | -0.05 | 0.05 | 0.985 |
| XL_VLDL_C_pct | 0.03 | -0.04 | 0.10 | 0.416 | -0.02 | -0.07 | 0.04 | 0.571 |
| XL_VLDL_CE_pct | 0.03 | -0.04 | 0.10 | 0.438 | -0.01 | -0.07 | 0.04 | 0.687 |
| XL_VLDL_FC_pct | 0.01 | -0.05 | 0.07 | 0.712 | -0.03 | -0.08 | 0.02 | 0.271 |
| XL_VLDL_TG_pct | -0.03 | -0.10 | 0.03 | 0.269 | 0.01 | -0.04 | 0.06 | 0.597 |
| L_VLDL_PL_pct | 0.01 | -0.04 | 0.07 | 0.671 | 0.04 | 0.00 | 0.08 | 0.049 |
| L_VLDL_C_pct | 0.03 | -0.02 | 0.09 | 0.258 | 0.01 | -0.03 | 0.05 | 0.684 |
| L_VLDL_CE_pct | 0.03 | -0.03 | 0.09 | 0.3 | 0.00 | -0.04 | 0.05 | 0.934 |
| L_VLDL_FC_pct | 0.04 | -0.02 | 0.10 | 0.221 | 0.02 | -0.02 | 0.07 | 0.309 |
| L_VLDL_TG_pct | -0.03 | -0.08 | 0.03 | 0.367 | -0.03 | -0.07 | 0.02 | 0.216 |
| M_VLDL_PL_pct | 0.04 | -0.01 | 0.08 | 0.121 | 0.03 | -0.01 | 0.06 | 0.157 |
| M_VLDL_C_pct | 0.04 | -0.01 | 0.09 | 0.148 | -0.01 | -0.06 | 0.03 | 0.538 |
| M_VLDL_CE_pct | 0.04 | -0.02 | 0.09 | 0.178 | -0.02 | -0.07 | 0.02 | 0.306 |
| M_VLDL_FC_pct | 0.04 | -0.01 | 0.08 | 0.127 | 0.01 | -0.03 | 0.05 | 0.616 |
| M_VLDL_TG_pct | -0.04 | -0.09 | 0.01 | 0.114 | 0.00 | -0.04 | 0.04 | 0.913 |
| S_VLDL_PL_pct | 0.04 | -0.01 | 0.09 | 0.142 | -0.01 | -0.06 | 0.03 | 0.519 |
| S_VLDL_C_pct | 0.04 | -0.01 | 0.09 | 0.128 | -0.01 | -0.05 | 0.03 | 0.743 |
| S_VLDL_CE_pct | 0.03 | -0.02 | 0.08 | 0.18 | 0.00 | -0.04 | 0.04 | 0.917 |
| S_VLDL_FC_pct | 0.04 | -0.01 | 0.09 | 0.157 | -0.02 | -0.06 | 0.03 | 0.46 |
| S_VLDL_TG_pct | -0.04 | -0.09 | 0.01 | 0.118 | 0.01 | -0.03 | 0.05 | 0.693 |
| XS_VLDL_PL_pct | -0.01 | -0.07 | 0.06 | 0.875 | -0.02 | -0.08 | 0.03 | 0.427 |
| XS_VLDL_C_pct | 0.00 | -0.06 | 0.06 | 0.907 | -0.05 | -0.10 | 0.00 | 0.034 |
| XS_VLDL_CE_pct | 0.00 | -0.06 | 0.06 | 0.953 | -0.05 | -0.10 | 0.00 | 0.046 |
| XS_VLDL_FC_pct | -0.02 | -0.08 | 0.05 | 0.63 | -0.05 | -0.10 | 0.00 | 0.065 |
| XS_VLDL_TG_pct | 0.00 | -0.05 | 0.06 | 0.887 | 0.06 | 0.02 | 0.11 | 0.004 |
| IDL_PL_pct | -0.02 | -0.09 | 0.04 | 0.505 | -0.01 | -0.06 | 0.05 | 0.812 |
| IDL_C_pct | 0.01 | -0.06 | 0.07 | 0.861 | -0.07 | -0.12 | -0.01 | 0.013 |
| IDL_CE_pct | 0.01 | -0.06 | 0.08 | 0.804 | -0.04 | -0.09 | 0.01 | 0.154 |
| IDL_FC_pct | -0.01 | -0.08 | 0.06 | 0.823 | -0.08 | -0.13 | -0.02 | 0.004 |
| IDL_TG_pct | 0.00 | -0.07 | 0.06 | 0.981 | 0.08 | 0.03 | 0.13 | 0.001 |
| L_LDL_PL_pct | -0.02 | -0.10 | 0.05 | 0.51 | -0.07 | -0.13 | -0.02 | 0.01 |
| L_LDL_C_pct | 0.01 | -0.05 | 0.08 | 0.682 | -0.03 | -0.08 | 0.02 | 0.203 |
| L_LDL_CE_pct | 0.04 | -0.02 | 0.11 | 0.223 | 0.04 | -0.02 | 0.09 | 0.17 |
| L_LDL_FC_pct | -0.04 | -0.10 | 0.03 | 0.257 | -0.10 | -0.15 | -0.05 | <0.001 |
| L_LDL_TG_pct | -0.01 | -0.07 | 0.05 | 0.734 | 0.07 | 0.02 | 0.12 | 0.004 |
| M_LDL_PL_pct | 0.03 | -0.04 | 0.10 | 0.465 | -0.07 | -0.12 | -0.01 | 0.016 |
| M_LDL_C_pct | 0.00 | -0.06 | 0.06 | 0.922 | -0.01 | -0.06 | 0.04 | 0.644 |
| M_LDL_CE_pct | 0.00 | -0.06 | 0.06 | 0.937 | 0.06 | 0.01 | 0.11 | 0.016 |
| M_LDL_FC_pct | 0.00 | -0.07 | 0.06 | 0.958 | -0.09 | -0.14 | -0.04 | <0.001 |
| M_LDL_TG_pct | -0.02 | -0.08 | 0.04 | 0.508 | 0.06 | 0.02 | 0.10 | 0.007 |
| S_LDL_PL_pct | -0.03 | -0.10 | 0.04 | 0.365 | -0.09 | -0.14 | -0.04 | 0.001 |
| S_LDL_C_pct | 0.03 | -0.03 | 0.09 | 0.348 | 0.02 | -0.03 | 0.06 | 0.516 |
| S_LDL_CE_pct | 0.03 | -0.04 | 0.09 | 0.417 | 0.09 | 0.04 | 0.14 | <0.001 |
| S_LDL_FC_pct | 0.01 | -0.06 | 0.08 | 0.796 | -0.11 | -0.16 | -0.06 | <0.001 |
| S_LDL_TG_pct | -0.02 | -0.08 | 0.05 | 0.637 | 0.10 | 0.05 | 0.15 | <0.001 |
| XL_HDL_PL_pct | -0.02 | -0.08 | 0.04 | 0.475 | -0.03 | -0.08 | 0.02 | 0.19 |
| XL_HDL_C_pct | 0.02 | -0.04 | 0.09 | 0.498 | -0.01 | -0.06 | 0.04 | 0.834 |
| XL_HDL_CE_pct | 0.06 | -0.01 | 0.14 | 0.093 | 0.00 | -0.06 | 0.06 | 0.998 |
| XL_HDL_FC_pct | -0.02 | -0.07 | 0.03 | 0.407 | -0.02 | -0.06 | 0.02 | 0.303 |
| XL_HDL_TG_pct | 0.01 | -0.06 | 0.08 | 0.822 | 0.13 | 0.07 | 0.18 | <0.001 |
| L_HDL_PL_pct | 0.01 | -0.05 | 0.06 | 0.735 | 0.03 | -0.02 | 0.07 | 0.247 |
| L_HDL_C_pct | 0.01 | -0.05 | 0.07 | 0.65 | -0.06 | -0.11 | -0.02 | 0.006 |
| L_HDL_CE_pct | 0.01 | -0.05 | 0.08 | 0.702 | -0.07 | -0.12 | -0.03 | 0.003 |
| L_HDL_FC_pct | 0.00 | -0.05 | 0.05 | 0.91 | 0.01 | -0.03 | 0.04 | 0.793 |
| L_HDL_TG_pct | -0.03 | -0.09 | 0.04 | 0.389 | 0.10 | 0.05 | 0.14 | <0.001 |
| M_HDL_PL_pct | -0.03 | -0.09 | 0.04 | 0.43 | 0.02 | -0.03 | 0.07 | 0.427 |
| M_HDL_C_pct | 0.03 | -0.04 | 0.09 | 0.442 | -0.06 | -0.11 | -0.01 | 0.014 |
| M_HDL_CE_pct | 0.02 | -0.05 | 0.09 | 0.525 | -0.08 | -0.13 | -0.03 | 0.003 |
| M_HDL_FC_pct | 0.02 | -0.03 | 0.07 | 0.448 | 0.04 | 0.00 | 0.08 | 0.085 |
| M_HDL_TG_pct | -0.02 | -0.09 | 0.05 | 0.539 | 0.09 | 0.04 | 0.14 | 0.001 |
| S_HDL_PL_pct | -0.03 | -0.10 | 0.03 | 0.284 | -0.02 | -0.07 | 0.03 | 0.421 |
| S_HDL_C_pct | 0.03 | -0.03 | 0.10 | 0.356 | -0.05 | -0.10 | 0.00 | 0.068 |
| S_HDL_CE_pct | 0.03 | -0.03 | 0.09 | 0.371 | -0.05 | -0.10 | -0.01 | 0.029 |
| S_HDL_FC_pct | 0.00 | -0.05 | 0.05 | 0.91 | 0.05 | 0.01 | 0.10 | 0.01 |
| S_HDL_TG_pct | -0.01 | -0.08 | 0.06 | 0.735 | 0.10 | 0.04 | 0.15 | <0.001 |

All models adjusted for baseline age, sex, duration of disease, baseline metabolite level, and country of recruitment. Increase over time exposure derived from residuals of final metabolite value regressed against baseline value.
